# Fasudil Protects Against Diclofenac-Induced Hepatorenal and Gastric Injury via Modulation of ROCK/TLR4/SIRT1 Signaling and Preservation of Tight Junctions

**DOI:** 10.3390/jox16040144

**Published:** 2026-08-02

**Authors:** Ali H. Al-Baldawi, Mahmoud M. Samaha, Marwa S. Zaghloul

**Affiliations:** 1 Department of Pharmacology and Toxicology, Faculty of Pharmacy, Mansoura University, Mansoura 35516, Egypt; baldawicosm79@gmail.com (A.H.A.-B.); msamaha@mans.edu.eg (M.M.S.); 2 Department of Pharmacology and Toxicology, Faculty of Pharmacy, Mansoura National University, Gamasa 7731168, Egypt

**Keywords:** fasudil, diclofenac, multi-organ NSAID toxicity, ROCK2/TLR4/SIRT1 pathway, TLR4, NF-κB

## Abstract

Background: Diclofenac is a widely consumed non-steroidal anti-inflammatory drug, yet its clinical utility is limited by severe hepatotoxicity, acute kidney injury, and gastric ulceration. While the RhoA/ROCK pathway is implicated in inflammation, its potential as a therapeutic target for multi-organ NSAID toxicity remains unexplored. Methods: This study evaluated the dose-dependent protective effects of the ROCK inhibitor, fasudil, against diclofenac-induced damage and investigated the underlying ROCK2/TLR4/SIRT1 axis. Five separate cohorts were established using thirty male Sprague-Dawley rats. Alongside a normal control and fasudil control group, one experimental group was treated with 100 mg/kg diclofenac. Furthermore, two distinct groups were pretreated with fasudil for seven days before induction, receiving either a 10 mg/kg or a 30 mg/kg dose prior to diclofenac administration. Results: Diclofenac provoked severe hepatic, renal, and gastric injury accompanied by marked oxidative stress. Pretreatment with fasudil markedly reversed these effects and improved hepatic and renal function biomarkers. Mechanistically, fasudil reduced renal and hepatic ROCK2 and TLR4 expression, which consequently suppressed downstream systemic inflammatory markers, including NF-κB and TNF-α. Furthermore, fasudil halted apoptosis by restoring SIRT1 expression and reducing cleaved caspase-3, alongside preserving gastric barrier integrity. Conclusions: Fasudil dose-dependently ameliorates diclofenac-induced hepatic, renal, and gastric injury by silencing the ROCK2/TLR4 inflammatory axis, restoring SIRT1 survival networks, and protecting epithelial tight junctions.

## 1. Introduction

As the most frequently utilized non-steroidal anti-inflammatory drug (NSAID) globally, diclofenac remains a primary therapeutic choice for managing pain and joint disorders such as osteoarthritis and rheumatoid arthritis [1]. However, recent clinical findings indicate that the administration of this medication carries significant health risks, most notably damage to the liver (hepatotoxicity), kidneys (nephrotoxicity), and stomach lining (gastric mucosal injury).

Epidemiological statistics indicate that utilizing NSAIDs elevates the likelihood of acute kidney injury (AKI) by 50.0% to 70.0%. Notably, diclofenac presents an eight-fold greater risk for renal complications when compared to alternative medications in this class [2,3]. Additionally, this medication represents a major catalyst for idiosyncratic drug-induced liver injury (DILI). In fact, epidemiological studies consistently identify it as the second most common pharmacological agent responsible for this condition [4]. Regarding gastrointestinal harm, NSAID administration accounts for 15.0% to 35.0% of peptic ulcer complications, with chronic usage leading to ulcer formation in up to 30.0% of patients [5]. Ultimately, these severe side effects represent a major international public health concern, highlighting the urgent necessity for preventative interventions.

The adverse effects of diclofenac across multiple organs arise from the dynamic interaction between sterile inflammation and oxidative stress, processes that are trackable through distinct molecular and biochemical markers. As the drug undergoes metabolic activation, reactive oxygen species (ROS) build up and induce lipid peroxidation, indicated by elevated malondialdehyde (MDA) alongside depleted levels of reduced glutathione (GSH) [6,7,8] w. This resulting oxidative imbalance functions as a primary catalyst for the thioredoxin-interacting protein/nucleotide-binding domain, leucine-rich–containing family, pyrin domain–containing-3 (TXNIP/NLRP3) inflammasome pathway, which subsequently initiates caspase-1-mediated pyroptosis [9,10]. Ultimately, this inflammatory cascade leads to severe organ impairment—detectable via elevation in creatinine, urea, and serum transaminases (AST, ALT) [6,11]—and compromises the epithelial barrier by suppressing essential tight junction proteins, including Zonula Occludens-1 (ZO-1) and claudins [12,13].

The nephrotoxic effects of diclofenac frequently manifest as AKI, spanning from minor functional impairment to complete renal failure [6]. The underlying pathophysiology involves suppressed prostaglandin synthesis due to cyclooxygenase (COX) inhibition, alongside mitochondrial oxidative damage and triggered inflammatory pathways. Consequently, patients experience substantial declines in glomerular filtration and the onset of tubular necrosis [14,15,16].

Regarding hepatic injury, diclofenac induces hepatocyte apoptosis primarily by interfering with mitochondrial and prostaglandin functions. This damage is compounded by the cyclic production of ROS, defective autophagy, and pro-apoptotic inflammatory signaling [17,18].

In the gastrointestinal tract, NSAID-related mucosal harm stems from halted prostaglandin production and the direct degradation of mitochondrial and epithelial cell membranes, a cascade that ends in mucosal bleeding, ulcer formation, and a compromised epithelial barrier [12,13,19]. Notably, diclofenac actively suppresses the expression of critical tight junction (TJ) proteins such as Occludin, ZO-1, and Claudins-1, -4, and -18 [12,13,19,20]. Because these proteins are vital for securing the gastric epithelial barrier, their downregulation increases mucosal permeability. This leakage provokes leukocyte infiltration, localized inflammation, and epithelial apoptosis, ultimately degrading the stomach’s protective lining [21,22,23].

Fasudil, a selective Rho-associated coiled-coil-containing protein kinase (ROCK) inhibitor, has garnered significant pharmacological interest due to its potent anti-inflammatory, anti-apoptotic, and antioxidant properties [24]. ROCK, a downstream effector of the small GTPase RhoA, plays a pivotal role in regulating the actin cytoskeleton, cellular adhesion, and motility [25]. Beyond cytoskeletal dynamics, the overactivation of the ROCK pathway is intimately linked to the exacerbation of oxidative stress and the amplification of pro-inflammatory signaling cascades. Mechanistically, ROCK activation facilitates the actin-driven degradation and internalization of essential TJ proteins, such as ZO-1 and Claudins, leading to compromised epithelial and endothelial barrier integrity; a hallmark of mucosal injury [26].

Furthermore, the RhoA/ROCK axis functions as a critical upstream regulator of the innate immune response and oxidative stress machinery. ROCK signaling promotes the activation of Toll-like receptor 4 (TLR4) and the subsequent nuclear translocation of nuclear factor kappa-B (NF-κB), thereby driving the robust expression of pro-inflammatory cytokines, including tumor necrosis factor alpha (TNF-α) and interleukin (IL)-1β [24,27]. This inflammatory priming is coupled with oxidative stress-mediated cellular damage, where ROCK activation upregulates TXNIP and triggers the assembly of the NLRP3 inflammasome [28]. The consequent activation of Caspase-1 and downstream executioner caspases, such as Cleaved Caspase-3, drives apoptotic cell death across various tissues. Conversely, Fasudil effectively interrupts this pathological cascade. By inhibiting ROCK, Fasudil not only represses the TLR4/NF-κB inflammatory and oxidative axis but also upregulates Sirtuin 1 (SIRT1), an NAD^+^-dependent histone deacetylase known for its profound cytoprotective and anti-apoptotic effects [29].

The present study is part of a broader research project investigating the protective effects of different pharmacological interventions against diclofenac-induced multi-organ toxicity. A related study from the same experimental cohort investigating the protective effects of oridonin against diclofenac-induced organ toxicity has been published previously [30]. To comply with the Reduction principle of the 3Rs, the normal and diclofenac groups were shared between both studies, whereas each manuscript investigated a different therapeutic compound and distinct molecular mechanisms.

The primary objective of this study is to evaluate the dose-dependent protective effects of fasudil (10 and 30 mg/kg) against diclofenac-induced hepatotoxicity, nephrotoxicity, and gastric injury in a rat model. Additionally, this study aims to elucidate the underlying molecular mechanisms, focusing on the modulation of the ROCK2/TLR4/SIRT1 signaling cascade, apoptotic markers, and epithelial barrier preservation.

## 2. Materials and Methods

### 2.1. Animals

Thirty male Sprague-Dawley rats (aged roughly 8 weeks, weighing 230 ± 30 g) were procured from VACSERA (the Holding Company for Biological Products and Vaccines) in Cairo, Giza, Egypt. Prior to the experimental procedures, the animals underwent a two-week acclimation phase in spacious cages under standard laboratory conditions at Mansoura University’s Faculty of Pharmacy animal facility. The present study is part of a broader research project its protocol received ethical clearance from both the Faculty of Pharmacy Research Ethics Committee and the Mansoura University Animal Care and Use Committee, under the approval ID: MU-ACUC (PHARM.MS.24.11.121). All procedures strictly adhered to the NIH Guide for the Care and Use of Laboratory Animals (Publication No. 85-23, revised 1985), the AVMA 2020 [31], and the ARRIVE 2.0 guidelines [32]. To comply with the Reduction principle of the 3Rs framework, common normal control and diclofenac-treated groups were shared across different treatment arms of the same ethically approved project. Therefore, these groups are identical to those reported in a related study derived from the same experimental cohort [30].

### 2.2. Drugs and Chemicals

Sigma-Aldrich (St. Louis, MO, USA) supplied the fasudil, which was prepared for oral dosing by suspension in a 0.5% *w*/*v* CMC-Na (sodium carboxymethylcellulose) vehicle. Additionally, the study utilized Novartis Pharma AG (Basel, Switzerland) as the source for diclofenac sodium, specifically employing their injectable Voltaren^®^ ampoules.

### 2.3. Experimental Work

Using a randomized complete block design, the animals were allocated into five distinct groups (*n* = 6 per group). The sample size was established using the resource equation method proposed by Charan and Kantharia (2013) [33], which is widely accepted and commonly applied in animal studies when reliable estimates of effect size are unavailable. The chosen group size was considered sufficient to identify the biologically meaningful effects while minimizing unnecessary animal use. The experimental treatments were administered as follows:

- Normal Control Group: Maintained on a 0.5% CMC-Na solution for seven consecutive days.
- Fasudil Control Group: This group received a seven-day regimen of fasudil (30 mg/kg), delivered strictly through a gastric tube.
- Diclofenac Group: Rats in this arm were given the 0.5% CMC-Na medium for seven days prior to receiving a single 100 mg/kg diclofenac sodium dose intraperitoneally on the eighth day [34,35].
- Low-Dose Fasudil (10 mg/kg) Group: Pretreated with 10 mg/kg fasudil via gastric gavage for seven days [36]. On day 8 (24 h post-pretreatment), rats were subjected to the standard diclofenac induction (100 mg/kg IP).
- High-Dose Fasudil (30 mg/kg) Group: Pretreated with 30 mg/kg fasudil via gastric gavage for seven days [36,37,38], succeeded by the identical diclofenac induction (100 mg/kg IP) on day 8.

Daily health assessments were executed by experienced personnel—familiar with normal rat physiology—to preemptively identify and mitigate any suffering. The experimental animals underwent routine monitoring for deviations in their baseline health, specifically focusing on water and feed consumption, weight fluctuations, postural or grooming abnormalities, and changes in locomotion or breathing rates. Data from any animal that contracted an unrelated infection or failed to receive the full pharmacological protocol due to technical failures during dosing, anesthesia, or biospecimen collection were strictly omitted from the final statistical analysis. Importantly, no animals met the exclusion criteria during the course of the study. Consequently, no animals were excluded from the analysis. Additionally, no animals died during the course of the experiment.

As the present study is a part of a broader research project and to comply with the Reduction principle of the 3Rs framework, common normal and diclofenac-treated groups were shared across different treatment arms of the same ethically approved project. Therefore, these groups are identical to those previously reported by Al-Baldawi et al. (2026) [30], which originated from the same experimental cohort. Specifically, the shared normal and diclofenac groups were reused solely to avoid unnecessary use of additional experimental animals.

### 2.4. Sample Collection

Immediately after administration of diclofenac on day 8, rats were individually housed in metabolic cages for urine collection for the following 24 h and subjected to water deprivation for the same period. This isolation facilitated the collection of pure urine by preventing admixture with food particles or feces. Upon completion of the collection timeframe, total urine volumes were measured. Each sample underwent a strict inspection for impurities, and any compromised specimens were excluded. Finally, the remaining uncontaminated urine aliquots were stored under strictly controlled temperature conditions pending further biochemical analysis [39,40].

On day 9, following completion of the 24 h urine collection period, rats were anesthetized with thiopental sodium (40 mg/kg, IP) [40]. Blood samples were subsequently drawn from the retro-orbital venous plexus utilizing capillary tubes. Following a period of coagulation at room temperature, the samples underwent centrifugation at 4000 rpm for 15 min to isolate the clear serum. This serum was then aliquoted and preserved at −80 °C pending future biochemical evaluations. Rats were followed by a 2-step euthanasia process; The initial anesthesia step involved thiopental sodium injection for serum collection, then exsanguination via cardiac puncture.

Following euthanasia and confirmation of death, the liver, kidneys, and stomach were promptly extracted and washed in chilled saline. The tissues were subsequently segregated based on experimental requirements:

- Histological and Immunohistochemical Analysis: A portion of the gastric body, the left kidneys, and the left hepatic lobes were preserved using a 10.0% formalin-buffered saline fixative.
- Biochemical Assessment: The right kidneys, right hepatic lobes, and residual stomach tissue were processed in ice-cold PBS using an Omni TH homogenizer (Omni International, Inc., Kennesaw, GA, USA) to yield a 10.0% (*w*/*v*) suspension.

Following homogenization, the biochemical samples underwent centrifugation at 4 °C and 4000 rpm (Sigma D-37520, Sigma Laborzentrifugen GmbH, Osterode am Harz Germany). The resulting clear supernatants were extracted and cryopreserved at −80 °C for future assays.

### 2.5. Quantification of Hepatorenal Biomarkers

Spectrophotometric analysis was employed to determine the serum concentrations of alanine aminotransferase (ALT) and aspartate aminotransferase (AST). These measurements were executed utilizing dedicated LiquiCHEK™ diagnostic kits (Agappe Diagnostics Limited; Pattimattom, Ernakulam, Kerala, India), specifically applying catalog numbers 11409005 and 11408005, respectively. All measurements were executed in accordance with the kinetic enzymatic protocols endorsed by the International Federation of Clinical Chemistry and Laboratory Medicine (IFCC) [41].

To determine the concentrations of serum and urinary creatinine, we utilized Creatinine-J reagent kits (Cat# MD1001111) based on the colorimetric kinetic Jaffé reaction [42]. Similarly, serum and urinary urea were quantified using UREA-LQ kits (Cat# TK41041), which employ an enzymatic UV kinetic assay driven by Urease and Glutamate Dehydrogenase (GLDH) [42]. Both assay kits were procured from SPINREACT, S.A. (Sant Esteve de Bas, Girona, Spain). All final absorbance readings were recorded utilizing a Labomed spectrophotometer (Labomed, Inc., Los Angeles, CA, USA).

To align with standardized clinical reporting metrics, all measured serum urea concentrations (mg/dL) were subsequently converted to blood urea nitrogen (BUN) values (mg/dL) by dividing the raw urea values by a stoichiometric conversion factor of 2.14, representing the molecular weight ratio of the entire urea molecule to its dual nitrogen atoms.

### 2.6. Total Protein Quantification

To quantify total protein content in tissue homogenates and urine samples, a colorimetric bicinchoninic acid (BCA) assay was performed using the BCA Protein Assay Kit II (Assay Genie, Dublin, Ireland, Cat#: BN01029) in accordance with the manufacturer’s protocol. The assay principle involves the reduction of cupric cations (Cu^+2^) to cuprous cations (Cu^+1^) by proteins in an alkaline environment, followed by the chelation of Cu^+1^ with BCA to form a water-soluble complex with a peak absorbance at 562 nm. Briefly, a standard curve was generated using bovine serum albumin (BSA) at concentrations ranging from 25 to 2000 µg/mL. The working reagent was prepared by mixing Reagent A and Reagent B in a 50:1 ratio. Subsequently, 25 µL of each standard and sample was incubated with 200 µL of the working reagent for 30 min at 37 °C. After cooling to room temperature, the absorbance was measured at 562 nm using a microplate reader. Total protein concentrations were then calculated based on the linear regression equation derived from the BSA standard curve using GraphPad Prism version 11.0.1 for Windows (GraphPad Software, Boston, MA, USA). This quantification served as the basis for normalizing target protein expression in Western blot analysis and evaluating urinary protein leakage.

### 2.7. Quantification of Oxidative Stress Markers in Homogenized Tissues

The concentrations of MDA, GSH, and total antioxidant capacity (TAC) within the kidney, liver, and stomach homogenates were evaluated calorimetrically. This analysis utilized specific commercial kits (Cat# MD2529 for MDA, Cat# GR2511 for GSH, and Cat# TA2513 for TAC) procured from Bio-Diagnostic (Giza, Egypt). All procedures were executed strictly following the protocols provided by the manufacturer. Final absorbance values were recorded with a Labomed spectrophotometer (Los Angeles, CA, USA).

### 2.8. Enzyme-Linked Immunosorbent Assay (ELISA)

The hepatic and renal concentrations of cluster of differentiation 68 (CD68), TNF-α, and ROCK2 were quantified utilizing species-specific rat ELISA kits. The specific commercial assays employed included Cat# MBS705029 from MyBioSource, Inc. (San Diego, CA, USA) for CD68, Cat# CSB-E11987r from CUSABIO (Wuhan, Hubei, China) for TNF-α, and Cat# ELK9932 from ELK Biotechnology Co., Ltd. (Wuhan, Hubei, China) for ROCK2.

To assess the concentrations of specific tight junction proteins within the gastric tissue, we utilized targeted ELISA kits. Specifically, ZO-1 and claudin-1 (CLDN1) levels were quantified using specialized assay kits (Cat# MBS706128 and Cat# MBS731496, respectively) procured from MyBioSource, Inc. (San Diego, CA, USA).

### 2.9. Western Blotting Analysis

Western blot analysis was employed to quantify the protein expression of SIRT1, TLR4, and cleaved caspase 3 within the kidney and liver tissues. Tissue homogenization for protein recovery was achieved utilizing a protease/phosphatase inhibitor-spiked RIPA lysis buffer (Bio Basic, Bio Basic Inc., Markham, ON, Canada; Cat# PL005). The mixtures were subjected to a 30 min ice bath, followed immediately by refrigerated centrifugation (4 °C) at roughly 16,000× *g* for 30 min. Finally, the separated supernatants were gathered to measure the total protein content with a colorimetric BCA Protein Assay Kit II (Assay Genie, Cat#: BN01029).

Prior to SDS-PAGE, 20 µg aliquots of each protein were diluted in a 1:1 ratio with a 2× Laemmli denaturing buffer. This mixture consisted of 10.0% 2-mercaptoethanol, 20.0% glycerol, 4.0% SDS, 0.004% bromophenol blue, and 0.125 M Tris-HCl (pH 6.8). After being subjected to a rolling boil for five minutes, the protein mixtures were resolved on Bio-Rad TGX Stain-Free™ FastCast™ acrylamide gels (Bio-Rad Laboratories, Inc., Hercules, CA, USA; Cat# 161-0181). The electrophoretic run commenced at 50 V for a 20 min stacking period, after which the voltage was elevated to 100–150 V until optimal resolution was attained. Following separation, the proteins were electroblotted onto PVDF membranes for 7 min at 25 V utilizing a Trans-Blot Turbo system (Bio-Rad Laboratories, Inc., Hercules, CA, USA). Finally, successful transfer was confirmed via stain-free imaging on a ChemiDoc™ imaging system (Bio-Rad Laboratories, Inc., Hercules, CA, USA).

To prevent non-specific binding, the membranes were blocked at room temperature for 1 h in a solution of 3.0% bovine serum albumin dissolved in TBST (150 mM NaCl, 0.1% Tween-20, 20 mM Tris-HCl, pH 7.5). Subsequently, the membranes were incubated overnight at 4 °C with specific primary antibodies prepared in the same blocking buffer. The applied primary antibodies included: mouse monoclonal anti-TLR4 (1–3 µg/mL; Thermo Fisher Scientific, Waltham, MA, USA; Cat# MA5-16216), rabbit polyclonal anti-cleaved caspase 3 (1:500–1:2000; Proteintech, Rosemont, IL, USA; Cat# 25128-1-AP), and mouse monoclonal anti-SIRT1 (1:500–1:2000; Thermo Fisher Scientific, Waltham, MA, USA; Cat# MA5-27217). To ensure equal protein loading, mouse monoclonal anti-β-actin (1:5000–1:20,000; Thermo Fisher Scientific, Waltham, MA, USA, Cat# MA1-140) was utilized as the internal control.

Post-incubation with the primary antibodies, the membranes underwent three to five 5 min washes in TBST. They were subsequently incubated at room temperature for 1 h with corresponding horseradish peroxidase-conjugated secondary antibodies. Following a final series of washes, the protein bands were detected utilizing Clarity™ Western ECL substrate (Bio-Rad, Bio-Rad Laboratories, Inc., Hercules, CA, USA, Cat# 170-5060). The generated chemiluminescence was detected using a ChemiDoc™ system (Bio-Rad Laboratories, Inc., Hercules, CA, USA), and subsequent densitometric analysis of the protein bands was performed with image processing software. Expression levels for the target markers were ultimately standardized against β-actin—acting as the endogenous reference—and the quantitative results were calculated as fold differences relative to the normal controls.

### 2.10. Morphological and Immunohistochemical Assessments

Following formalin fixation, the tissue specimens underwent dehydration via a graded series of ethanol, clearing with xylene, and subsequent embedding in paraffin wax. Using a microtome, these paraffin blocks were sectioned at a thickness of 5 µm. The resulting tissue slices were stained with hematoxylin and eosin (H&E) and examined using an Olympus CH2 light microscope (Olympus Corporation, Hachioji, Tokyo, Japan). To ensure unbiased histopathological evaluation, all slides were pre-coded for a blinded analysis, with four distinct sections assessed per slide for every animal. Selection of representative microscopic fields was carried out from multiple randomly chosen non-overlapping tissue sections from fields that reflected the overall histopathological features observed within each tissue section in each animal to ensure adequate representation of the overall histopathological alterations.

To quantify the extent of gastric mucosal damage, we utilized a modified four-point histopathological scoring framework [43]. Under microscopic examination, tissues were evaluated for four distinct pathological features: necrosis, ulceration, erosion, and edema. Each parameter was scored on a binary scale, receiving a 1 if present or a 0 if absent. A cumulative histopathological score for each sample was then calculated by aggregating these individual values. For every experimental group, this analysis was conducted across six randomly chosen microscopic fields. To quantify hepatic injury, a semi-quantitative grading scale from 0 to 4 was utilized: a score of 0 indicated normal architecture, 1 represented minimal lesions, 2 denoted mild lesions, 3 indicated moderate lesions, and 4 was assigned for severe tissue damage [44]. For the renal histopathological evaluation, analysis was strictly restricted to the renal cortex to effectively score both glomerular and tubular damage. Tubular injury—identified by epithelial cell sloughing, hydropic degeneration, tubular necrosis, and dilation—was graded based on the proportion of affected tubules: 0 (no injury), 1 (<10.0%), 2 (10.0–25.0%), 3 (26.0–50.0%), 4 (51.0–75.0%), and 5 (>75.0%) [45]. Concurrently, glomerular damage was evaluated on a 0 to 3 scale, assigning 0 for normal structural integrity, 1 for Bowman’s capsule thickening, 2 for glomerular tuft retraction, and 3 for the presence of glomerular fibrosis [46].

To evaluate the expression of interleukin-1β (IL-1β) and nuclear factor kappa-B (NF-κB) within kidney and liver tissues, we performed immunostaining via the Avidin-Biotin Complex technique [47]. Following the deparaffinization and rehydration of the tissue sections, high-pressure antigen retrieval was conducted using a 10 mM citrate buffer (pH 6.0), adhering to the specific requirements for the primary antibodies. To execute the immunostaining, tissue specimens were exposed to a panel of rabbit polyclonal primary antibodies. The protocol utilized anti-occludin (Cat# A2601; ABclonal, Woburn, MA, USA), anti-IL-1β at a 1:800 dilution (Cat# GB11113; ServiceBio, Wuhan, China), and anti-NF-κB p65/RelA diluted 1:200 (Cat# A2547; ABclonal). Microscopic evaluation was conducted using a Japanese-manufactured Olympus CH2 light microscope. To ensure absolute objectivity, all slides were pre-coded, allowing the investigator to review them without knowledge of the treatment groups. The final data points generated for statistical analysis represented the arithmetic mean of four independent sections analyzed from each subject’s slide.

For high-resolution image analysis, whole-tissue sections were processed using ImageJ (FIJI 2.10.0, National Institutes of Health, MD, USA). To quantify the relative antigen content, the region of interest was virtually extracted from sections at magnifications reaching 40×. Color deconvolution was applied to extract the specific brown DAB chromogen from the surrounding hematoxylin staining. Subsequently, the generated monochrome DAB images were subjected to quantitative analysis utilizing ImageJ software (FIJI, NIH, USA) to assess the antigen’s area fraction. The final expression values for both the hepatic and renal samples represent the proportion of the immunopositive region, with all data presented as means ± SD [48].

### 2.11. Statistical Analysis

Statistical evaluations were performed using GraphPad Prism version 11.0.1 for Windows (GraphPad Software, Boston, MA, USA). Data normality was first verified via the Shapiro–Wilk test [49]. For datasets meeting the criteria for normality, a one-way ANOVA followed by Tukey’s post hoc test for multiple comparisons was utilized. When normal distribution criteria were not met, statistical evaluations were carried out via the Kruskal–Wallis test, and Dunn’s approach was utilized for all subsequent between-group comparisons. The final quantitative values are summarized as means ± SD. An alpha level of ≤0.05 (two-tailed) was adopted to denote statistical significance.

## 3. Results

The fasudil control group did not differ significantly from the normal control group in any of the evaluated parameters. Therefore, subsequent comparisons focused primarily on the diclofenac-induced changes relative to the control groups and the effects of fasudil treatment.

### 3.1. Fasudil Mitigated Diclofenac-Evoked Impairments in Renal and Hepatic Function Biomarkers

#### 3.1.1. Urinary Kidney Function Biomarkers

Diclofenac intoxication markedly altered urinary biochemical parameters. This was characterized by a precipitous 80.0% decline in urine creatinine level compared to the normal control group (*p* < 0.0001). Furthermore, diclofenac induced marked proteinuria, elevating urine total protein by 372.4% over baseline levels (*p* < 0.0001). Treatment with fasudil effectively counteracted these urinary abnormalities. Fasudil at 10 mg/kg increased urine creatinine levels by 69.7% compared with the diclofenac group; this difference did not reach statistical significance (*p* = 0.0866). In contrast, the 30 mg/kg dose produced a significant 221.9% increase in urine creatinine levels compared with the diclofenac group (*p* < 0.0001).

**Figure 1:**
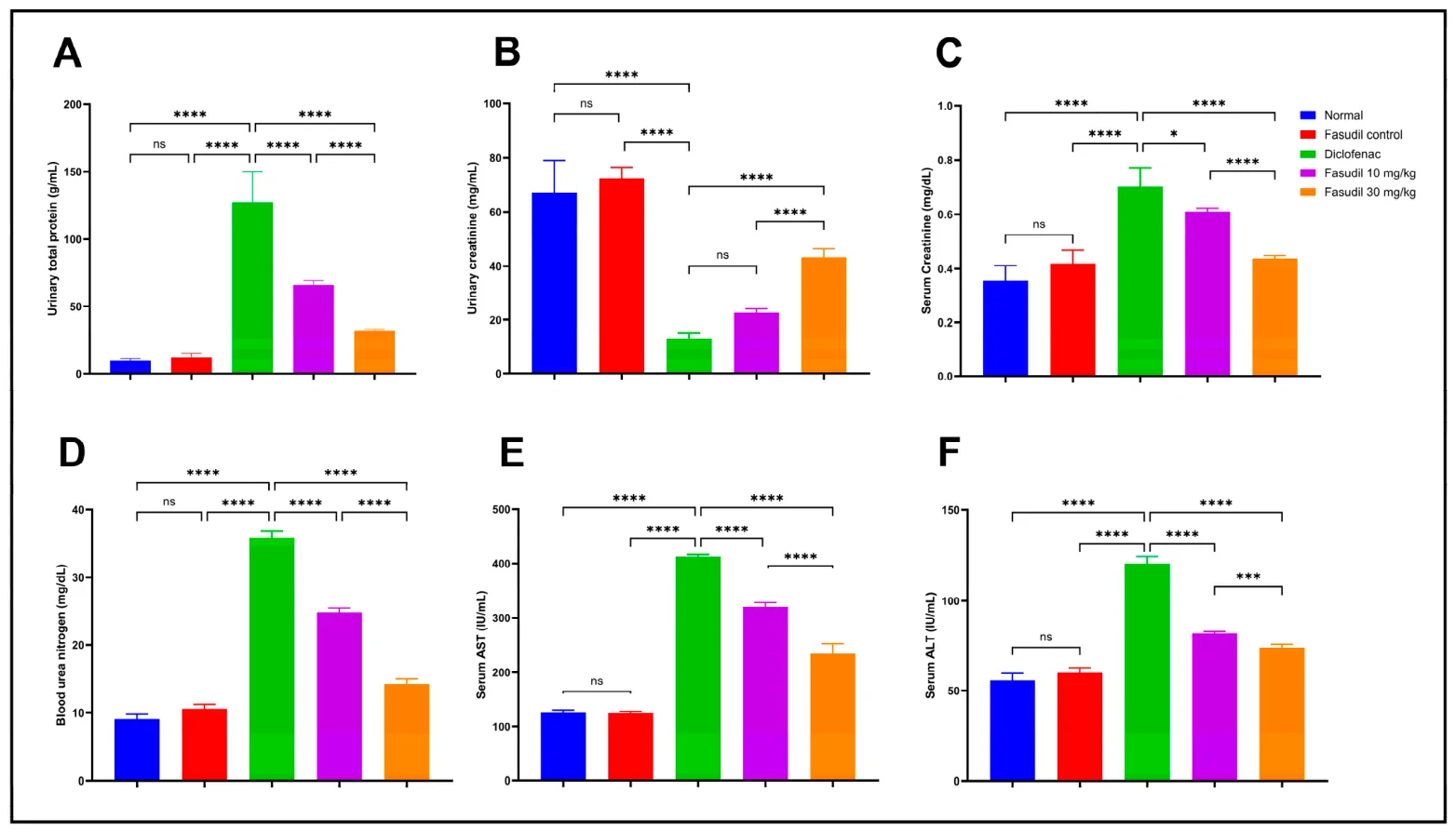
Fasudil attenuates diclofenac-induced dysfunction in hepatorenal biomarkers. The panels illustrate urine analyses for (**A**) total protein and (**B**) creatinine, alongside systemic serum measurements for (**C**) creatinine, (**D**) blood urea nitrogen, (**E**) AST, and (**F**) ALT. Values represent the mean ± standard deviation (*n* = 6 animals per cohort). The treatment parameters consisted of a solitary intraperitoneal diclofenac injection (100 mg/kg) and a one-week oral regimen of fasudil (at either 10 or 30 mg/kg). Between-group variance was assessed utilizing a one-way analysis of variance (ANOVA), coupled with Tukey’s post hoc procedure for multiple comparisons. Statistical significance is indicated by asterisks: * *p* < 0.05, *** *p* < 0.001, and **** *p* < 0.0001. Abbreviations: ALT, alanine aminotransferase; AST, aspartate aminotransferase. The Normal and Diclofenac data presented in this figure were previously reported by Al-Baldawi et al. (2026) [30] and are reused here because both studies originated from the same ethically approved experimental cohort.

Simultaneously, fasudil suppressed total urinary protein leakage in a highly significant manner. Compared with the diclofenac group, total urine protein was reduced by 30.6% in the 10 mg/kg group and by 60.3% in the 30 mg/kg group (both *p* < 0.0001). Interestingly, our results showed that fasudil 30 mg/kg had a more protective effect than 10 mg/kg, as evidenced by a significant decrease in urinary total protein and a significant increase in urinary creatinine levels (Figure 1A,B).

#### 3.1.2. Serum Kidney Function Biomarkers

The administration of diclofenac (100 mg/kg) provoked a profound deterioration in renal function. This was evidenced by a massive 1202% increase in serum blood urea nitrogen (BUN) levels compared with the normal control group (*p* < 0.0001). Similarly, serum creatinine levels were elevated by 97.2% in the diclofenac-intoxicated rats compared to the normal controls (*p* < 0.0001). Compared with the diclofenac group, fasudil at 10 mg/kg significantly reduced BUN levels by 48.0% (*p* < 0.0001). This nephroprotective effect was markedly enhanced at the higher fasudil dose (30 mg/kg), resulting in a 74.7% reduction in BUN compared with the diclofenac group (*p* < 0.0001). For serum creatinine, treatment with the lower dose (10 mg/kg) resulted in a significant 12.9% reduction (*p* = 0.0423), whereas the 30 mg/kg dose robustly and significantly suppressed the creatinine elevation, reducing it by 41.8% compared to the diclofenac group (*p* < 0.0001). Interestingly, fasudil 30 mg/kg showed a more protective effect than 10 mg/kg, as reflected by significant decreases in serum creatinine and BUN levels (Figure 1C,D).

#### 3.1.3. Serum Liver Function Biomarkers

The administration of diclofenac (100 mg/kg) provoked a profound deterioration in liver function and hepatocellular integrity. This was evidenced by a 230.5% increase in serum AST activity compared with the normal control group (*p* < 0.0001). Similarly, serum ALT levels were significantly elevated by 116.8% in the diclofenac-intoxicated rats compared to the normal controls (*p* < 0.0001). Compared with the diclofenac group, administration of fasudil at 10 mg/kg provided a significant hepatoprotective effect, reducing serum AST levels by 22.1% and ALT levels by 32.0% (both *p* < 0.0001). This protective efficacy was markedly enhanced at the higher fasudil dose (30 mg/kg), which yielded a robust 43.3% reduction in serum AST and a 38.2% reduction in serum ALT relative to the diclofenac group (both *p* < 0.0001). Furthermore, the 30 mg/kg dose demonstrated significantly superior restorative effects compared to the 10 mg/kg dose across both liver enzymes (Figure 1E,F).

### 3.2. Fasudil Abrogated Diclofenac-Evoked Oxidative Stress

**Figure 2:**
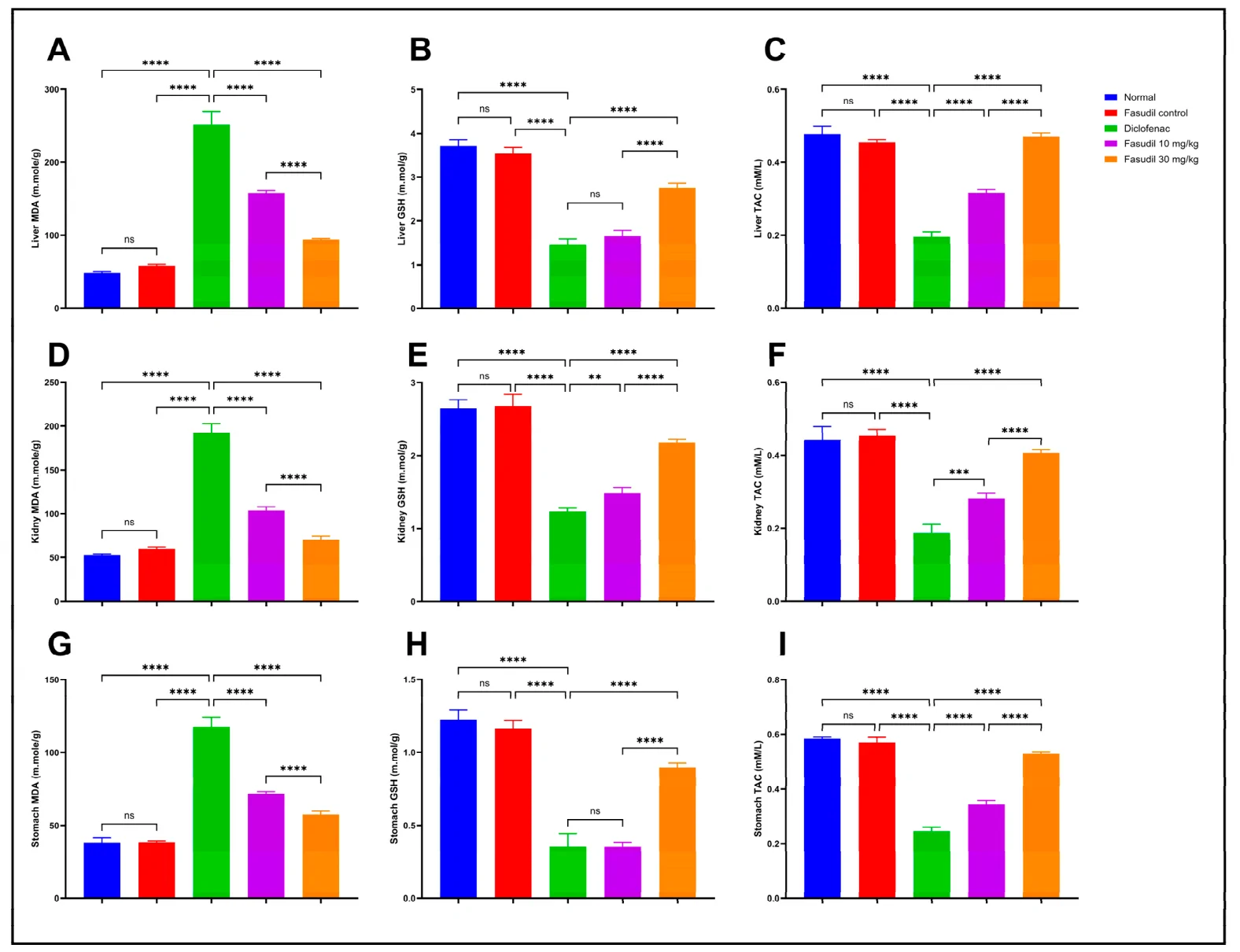
Suppression of diclofenac-induced oxidative damage following fasudil administration. The panels depict redox parameters across three distinct tissues: hepatic concentrations of (**A**) MDA, (**B**) GSH, and (**C**) TAC; renal measurements of (**D**) MDA, (**E**) GSH, and (**F**) TAC; and gastric levels of (**G**) MDA, (**H**) GSH, and (**I**) TAC. Findings are plotted as means with their corresponding standard deviations for cohorts of six subjects. The administration protocols utilized a one-time IP dose of diclofenac (100 mg/kg) and daily oral fasudil (10 or 30 mg/kg) over a seven-day period. To assess statistical variance, a one-way ANOVA paired with Tukey’s multiple comparison method was applied, where ** indicates *p* < 0.01, *** indicates *p* < 0.001, and **** indicates *p* < 0.0001. TAC = total antioxidant capacity; MDA = malondialdehyde; GSH = reduced glutathione. The Normal and Diclofenac data presented in this figure were previously reported by Al-Baldawi et al. (2026) [30] and are reused here because both studies originated from the same ethically approved experimental cohort.

To elucidate the systemic impact of the fasudil treatments on diclofenac-induced oxidative damage, the levels of MDA—a primary marker of lipid peroxidation—alongside endogenous antioxidant defenses, including GSH and TAC, were quantified across renal, hepatic, and gastric tissue homogenates (Figure 2).

#### 3.2.1. Hepatic Tissue Homogenate

Administration of diclofenac (100 mg/kg) induced severe oxidative stress, characterized by a profound 420.3% increase in hepatic MDA levels relative to the normal control group (*p* < 0.0001). This was accompanied by a 60.6% decline in hepatic GSH and a 59.2% reduction in TAC (both *p* < 0.0001). Pretreatment with fasudil effectively neutralized this hepatic oxidative burden. The 10 mg/kg dose significantly reduced hepatic MDA by 37.3% and induced a remarkable 141.2% restoration in TAC (both *p* < 0.0001), though its effect on GSH was not statistically significant (*p* = 0.1219). However, the higher fasudil dose (30 mg/kg) effectively restored hepatic oxidant/antioxidant balance, yielding a 62.6% reduction in MDA accumulation, an 89.0% increase in GSH, and a 62.2% increase in TAC compared with the diclofenac group (all *p* < 0.0001). Interestingly, comparative analysis revealed that fasudil at 30 mg/kg exerted significantly greater efficacy than the 10 mg/kg dose in attenuating MDA levels and enhancing antioxidant defenses in liver tissue homogenates (Figure 2A–C).

#### 3.2.2. Renal Tissue Homogenate

Administration of diclofenac (100 mg/kg) induced severe oxidative stress. This was evidenced by a significant 262.2% increase in kidney MDA levels relative to the normal control group (*p* < 0.0001). Concurrently, diclofenac intoxication severely depleted the kidneys’ innate antioxidant reservoirs, leading to a precipitous 48.2% decline in GSH levels and a 57.9% reduction in TAC compared to normal controls (both *p* < 0.0001). Pretreatment with fasudil successfully counteracted this oxidative imbalance in a dose-dependent manner. Compared to the diclofenac group, administration of fasudil at 10 mg/kg significantly suppressed lipid peroxidation by reducing renal MDA levels by 46.0% (*p* < 0.0001), while simultaneously boosting GSH by 20.3% (*p* = 0.0017) and TAC by 50.3% (*p* < 0.0001). This antioxidant efficacy was remarkably enhanced at the 30 mg/kg dose, yielding a robust 63.3% reduction in MDA accumulation, a 76.3% elevation in GSH, and a marked 118.1% increase in TAC (all *p* < 0.0001). Within the processed renal tissue, the higher fasudil dosage (30 mg/kg) markedly outperformed the lower dosage (10 mg/kg) by producing a statistically greater reduction in MDA expression alongside a more robust enhancement of the local antioxidant profile (Figure 2D–F).

#### 3.2.3. Gastric Tissue Homogenate

Furthermore, the gastric mucosa suffered severe oxidative derangements following diclofenac intoxication. Gastric MDA levels elevated by 207.9% over normal baseline values (*p* < 0.0001), while critical mucosal antioxidants plummeted, reflecting a 63.2% loss in GSH and a 58.0% depletion in TAC (both *p* < 0.0001). Compared with the diclofenac group, the 10 mg/kg dose lowered gastric MDA by 39.4% and improved TAC by 40.6% (both *p* < 0.0001), though it did not significantly alter GSH levels (*p* = 0.4171). Pretreatment with fasudil (30 mg/kg), however, demonstrated profound mucosal protection: it suppressed gastric lipid peroxidation by 51.2%, almost entirely restored GSH pools with a 99.0% relative increase, and induced a 115.8% elevation in TAC (all *p* < 0.0001). Crucially, within the homogenized stomach tissues, the higher fasudil concentration (30 mg/kg) significantly outperformed the lower dosage (10 mg/kg). This greater potency was evidenced by a more substantial suppression of MDA levels paired with a markedly improved local antioxidant profile (Figure 2G–I).

### 3.3. Fasudil Suppressed Diclofenac-Evoked ROCK2 Overactivity in Hepatic and Renal Tissues

**Figure 3:**
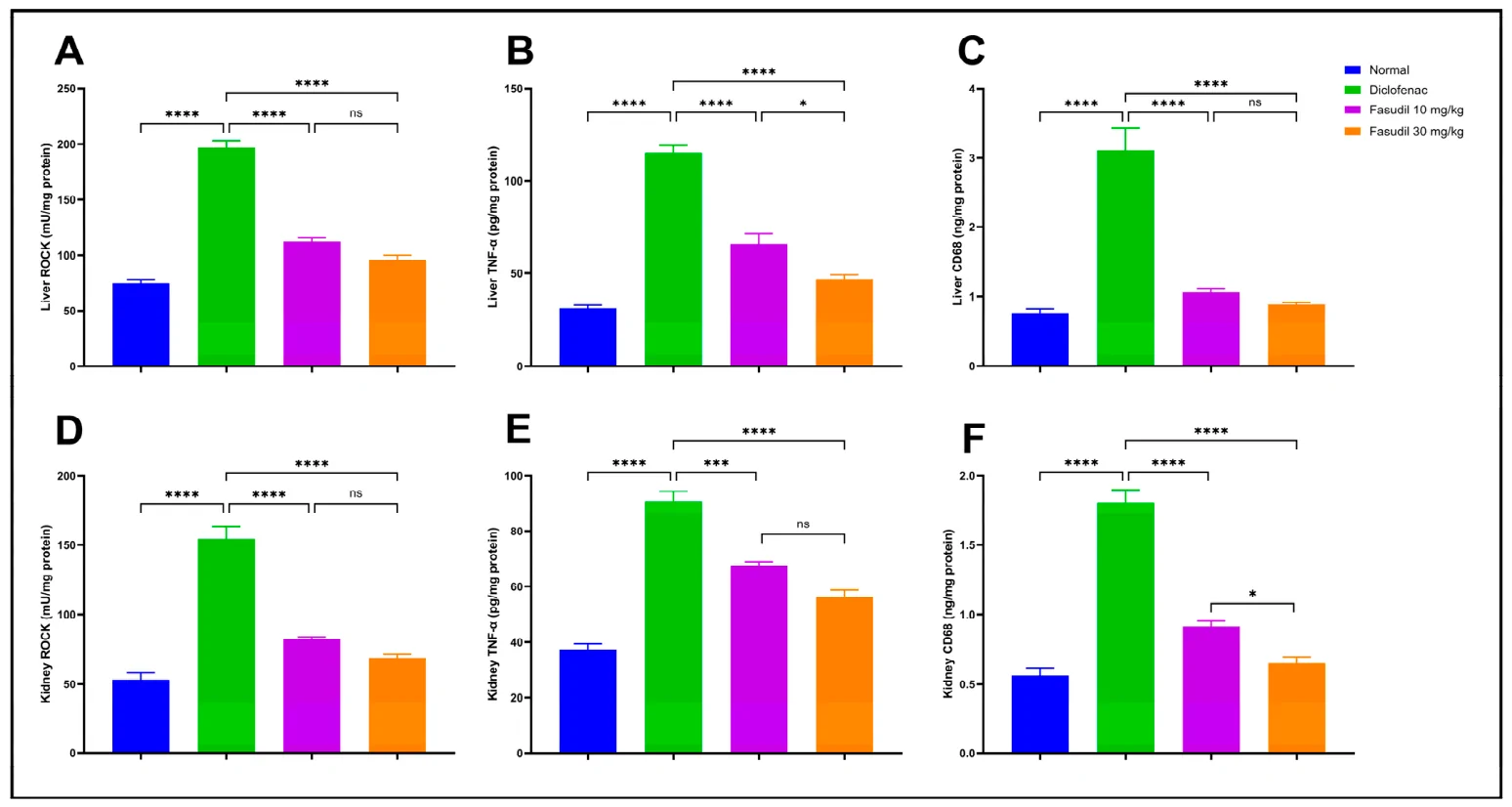
Fasudil downregulates diclofenac-induced ROCK2 hyperactivation and inflammatory markers across hepatorenal tissues. The panels illustrate hepatic expression profiles for (**A**) ROCK2, (**B**) the pro-inflammatory cytokine TNF-α, and (**C**) the macrophage marker CD68. Corresponding renal assessments are displayed for (**D**) ROCK2, (**E**) TNF-α, and (**F**) CD68. Results represent the mean ± SD (*n* = 4). Treatment conditions included a one-time 100 mg/kg IP diclofenac exposure and a daily oral fasudil pretreatment (10 or 30 mg/kg) spanning one week. Statistical outcomes were computed via a one-way ANOVA paired with Tukey’s test for multiple comparisons. Asterisks indicate established alpha levels (* *p* < 0.05, *** *p* < 0.001, **** *p* < 0.0001). CD68: Cluster of Differentiation 68; ROCK2: Rho-associated coiled-coil-containing protein kinase 2; TNF-α: Tumor Necrosis Factor-alpha.

To definitively establish that the multi-organ protection afforded by fasudil was driven by the inhibition of its primary pharmacological target, the protein levels of ROCK2 were quantified in both hepatic and renal tissues. The systemic toxicity induced by diclofenac (100 mg/kg) was accompanied by a marked pathological activation of the ROCK2 signaling pathway. In the renal microenvironment, diclofenac intoxication provoked a profound 190.0% elevation in ROCK2 levels relative to the normal control group (*p* < 0.0001). Similarly, in the liver, diclofenac exposure induced a 165.6% increase in hepatic ROCK2 levels relative to baseline (*p* < 0.0001). This confirms that ROCK2 hyperactivation is a central downstream mechanism of diclofenac-induced injury (Figure 3A–D).

Pretreatment with the ROCK inhibitor, fasudil, effectively dismantled this pathogenic signaling cascade across both organ systems. Compared with the diclofenac group, fasudil at 10 mg/kg significantly suppressed target levels, reducing ROCK2 levels by 46.9% in the kidneys and 43.1% in the liver (both *p* < 0.0001). Pretreatment with fasudil 30 mg/kg yielded 55.5% and 51.3% reductions in renal and hepatic ROCK2 levels, respectively, relative to the diclofenac group (*p* < 0.0001). Numerically, the 30 mg/kg dose showed a greater reduction than the 10 mg/kg dose; however, the difference between the two doses was not statistically significant in renal (*p* = 0.3581) and hepatic tissues (*p* = 0.1130). Most importantly, statistical comparisons revealed no significant difference in renal ROCK2 levels between the 30 mg/kg fasudil group and healthy normal controls (*p* = 0.2360), indicating that the higher fasudil dose completely suppressed the diclofenac-induced ROCK2 elevation and restored target levels to healthy, homeostatic baseline levels. Ultimately, these results provide compelling molecular evidence that fasudil’s organ-protective, anti-inflammatory, and anti-apoptotic effects are associated with modulation of the ROCK2 signaling axis (Figure 3A–D).

### 3.4. Fasudil Suppressed Diclofenac-Evoked Inflammation and TLR4 Signaling in Hepatic and Renal Tissues

To elucidate the anti-inflammatory mechanisms underlying the organ-protective effects of fasudil, the downstream pro-inflammatory markers (TNF-α), macrophage infiltration markers (CD68), and the expression of key inflammatory mediators (NF-κB and IL-1β), as well as TLR4, were evaluated in both hepatic and renal tissues.

#### 3.4.1. Pro-Inflammatory and Macrophage Infiltration Markers (TNF-α and CD68)

In the renal microenvironment, diclofenac intoxication resulted in a 143.0% increase in TNF-α levels and a 220.5% increase in renal CD68 levels relative to the normal control group (both *p* < 0.0001). A correspondingly pronounced inflammatory response was observed in hepatic tissues, where diclofenac exposure was accompanied by a substantial 268.4% increase in hepatic TNF-α levels and a 250.5% increase in macrophage-specific CD68 levels compared to normal controls (both *p* < 0.0001) (Figure 3B,C,E,F).

Compared with the diclofenac group, administration of fasudil at 10 mg/kg significantly decreased TNF-α levels by 25.7% and 42.9% in the kidney (*p* = 0.0001) and liver (*p* < 0.0001), respectively, and successfully attenuated macrophage infiltration by reducing CD68 levels by 49.1% and 60.2% in the kidney and liver, respectively (both *p* < 0.0001). The anti-inflammatory efficacy of fasudil was remarkably enhanced at the 30 mg/kg dose, which significantly attenuated the inflammatory signaling, mitigating renal and hepatic TNF- α levels by 38.1% and 59.4%, respectively (both *p* < 0.0001). Furthermore, the 30 mg/kg pretreatment effectively halted inflammatory cell recruitment, precipitating 64.0% decline in renal CD68 levels and a 66.6% decline in hepatic CD68 levels relative to the diclofenac group (both *p* < 0.0001) (Figure 3B,C,E,F).

#### 3.4.2. Inflammatory Mediators (NF-κB and IL-1β)

**Figure 4:**
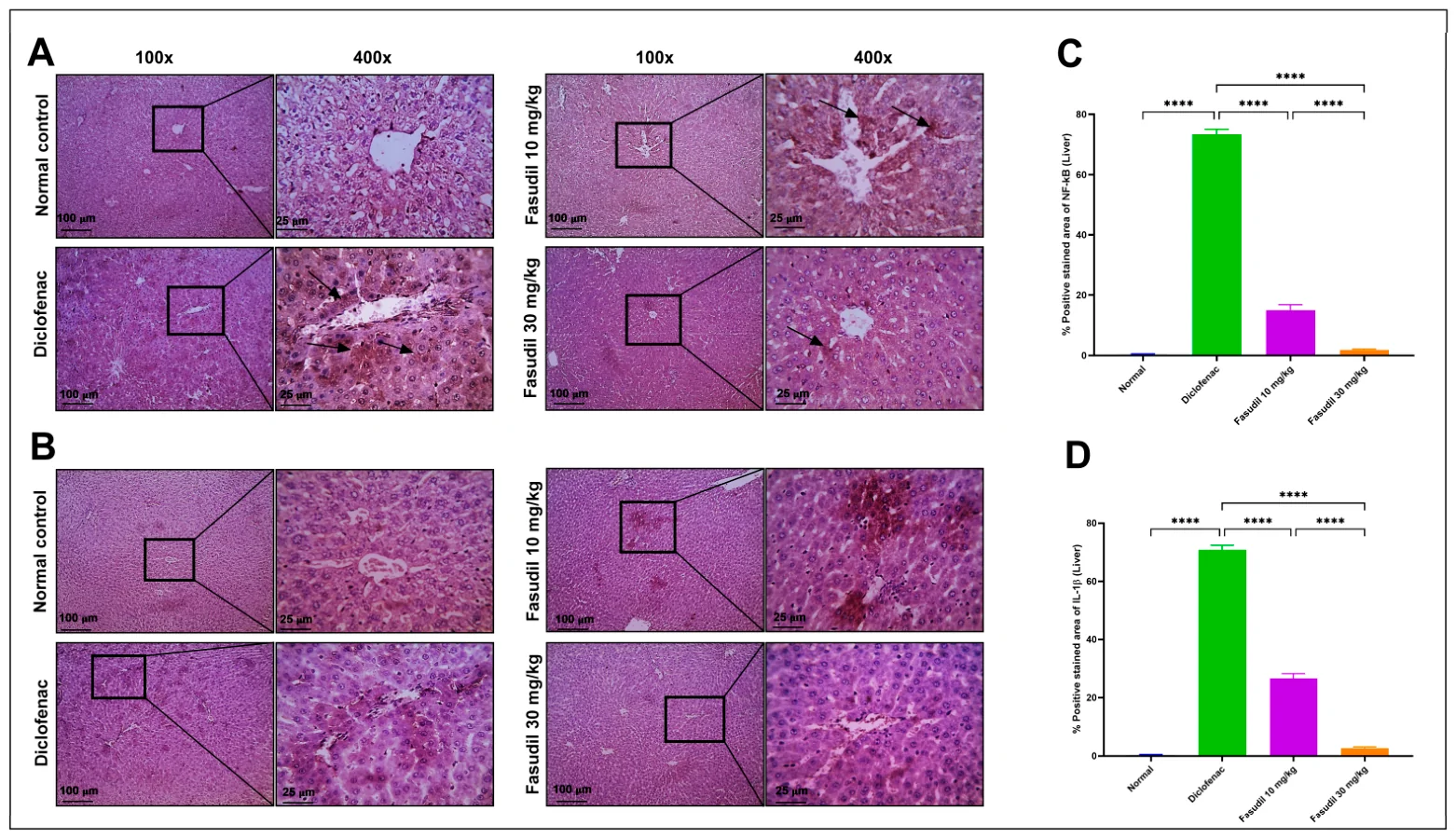
Fasudil suppressed diclofenac evoked-hepatic inflammatory mediators (NF-κB and IL-1β). (**A**,**B**) Representative immunohistochemical images of liver sections stained for (**A**) NF-κB and (**B**) IL-1β. (**C**,**D**) Histograms illustrating the percentage of positively stained areas for (**C**) NF-κB and (**D**) IL-1β expression. Black arrows: strong positive brown staining for both NF-κB and IL-1β in numerous hepatocytes. Mayer’s hematoxylin was applied as a counterstain for all IHC slides. Representative micrographs are displayed at 100× and 400× magnifications, corresponding to 100 μm and 25 μm scale bars, respectively. All data points reflect the mean ± SD (*n* = 6). The in vivo regimen involved delivering fasudil (10 or 30 mg/kg) via oral gavage for seven consecutive days, immediately followed by a one-time IP diclofenac dose (100 mg/kg) on the eighth day. Statistical differences were resolved by employing a one-way ANOVA integrated with Tukey’s test for multiple comparisons (**** *p* < 0.0001). Original images are provided in the Supplementary Materials.

**Figure 5:**
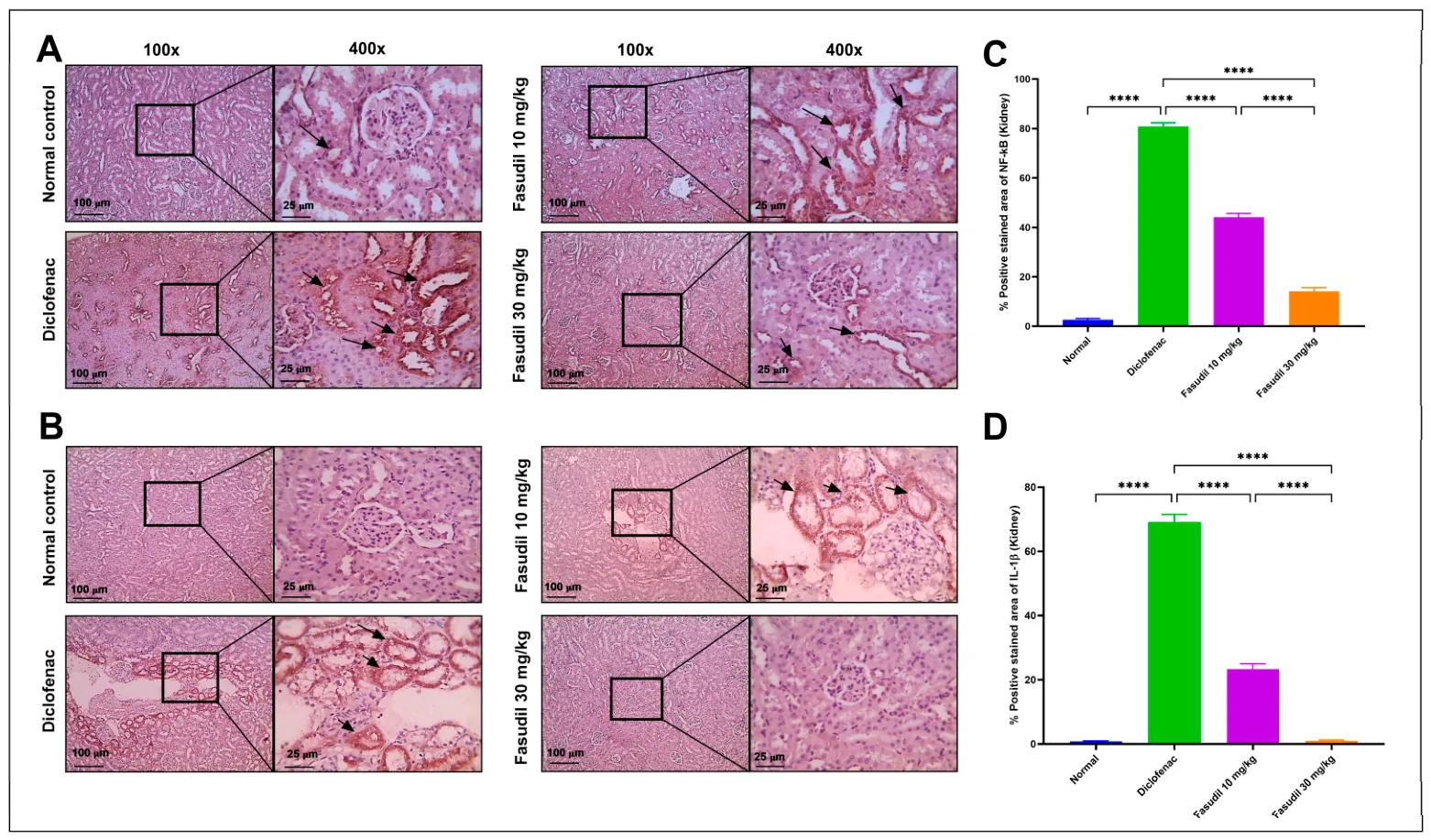
Mitigation of the diclofenac-associated upregulation of IL-1β and NF-κB in renal tissues by fasudil. Microscopic evaluations are depicted via representative IHC images for (**A**) NF-κB and (**B**) IL-1β in the kidney. The graphical data presented in (**C**,**D**) summarize the relative percentage of positively stained areas for each respective target. Black arrows denote regions of heavy brown staining, reflecting the high presence of NF-κB and IL-1β in multiple hepatocytes. Immunostained tissues were counterstained with Mayer’s hematoxylin and imaged at 100× (100 μm bar) and 400× (25 μm bar). Results are given as means ± SD (*n* = 6). The dosing schedule consisted of a 7-day fasudil pretreatment (10 or 30 mg/kg, orally) prior to a single diclofenac injection (100 mg/kg, IP) on day eight. Differences were analyzed by one-way ANOVA and Tukey’s test for multiple comparisons, with **** indicating *p* < 0.0001. Original images are provided in the Supplementary Materials.

In the control group, liver and kidney tissues exhibited virtually negative staining for both markers. In the diclofenac group, a robust inflammatory response was observed, characterized by strong positive brown expression of NF-κB and IL-1β in numerous hepatocytes and renal tubules. Quantitative image analysis confirmed that the diclofenac group showed a significant increase in NF-κB and IL-1β expression compared with the control group (both *p* < 0.0001) (Figure 4 and Figure 5).

In liver tissues, treatment with 10 mg/kg fasudil visibly decreased positive staining and significantly reduced NF-κB expression by 79.5% and IL-1β expression by 62.4% relative to the diclofenac group (both *p* < 0.0001). Notably, 30 mg/kg fasudil further attenuated inflammation, resulting in only scattered NF-κB positivity and complete absence of IL-1β staining. Quantitative evaluation revealed that 30 mg/kg fasudil substantially mitigated the diclofenac-induced inflammation, reducing NF-κB by 97.5% and IL-1β by 96.2% compared to the diclofenac group (both *p* < 0.0001). This dose effectively normalized the expression of these markers to levels not significantly different from those of the healthy control group (*p* = 0.8307 for NF-κB, *p* = 0.5042 for IL-1β). Collectively, these findings demonstrate that fasudil potently mitigated hepatic inflammation (Figure 4 and Figure 5).

In kidney tissues, treatment with 10 mg/kg fasudil visibly decreased positive staining in some tubules, significantly reducing NF-κB expression by 45.4% and IL-1β expression by 66.3% relative to the diclofenac group (both *p* < 0.0001). Notably, 30 mg/kg fasudil further attenuated inflammation, resulting in only mild positive NF-κB staining in a few tubules and completely negative IL-1β staining. Quantitative evaluation revealed that 30 mg/kg fasudil drastically reduced diclofenac-induced inflammation, dropping NF-κB by 82.5% and IL-1β by 98.6% compared to the diclofenac group (both *p* < 0.0001). While the 30 mg/kg dose effectively normalized IL-1β expression to levels not significantly different from those of the healthy control group (*p* = 0.9998), NF-κB expression was markedly improved but remained above baseline levels (*p* < 0.0001). Collectively, these findings demonstrate that fasudil potently mitigated renal inflammation (Figure 4 and Figure 5).

#### 3.4.3. TLR4 Expression

**Figure 6:**
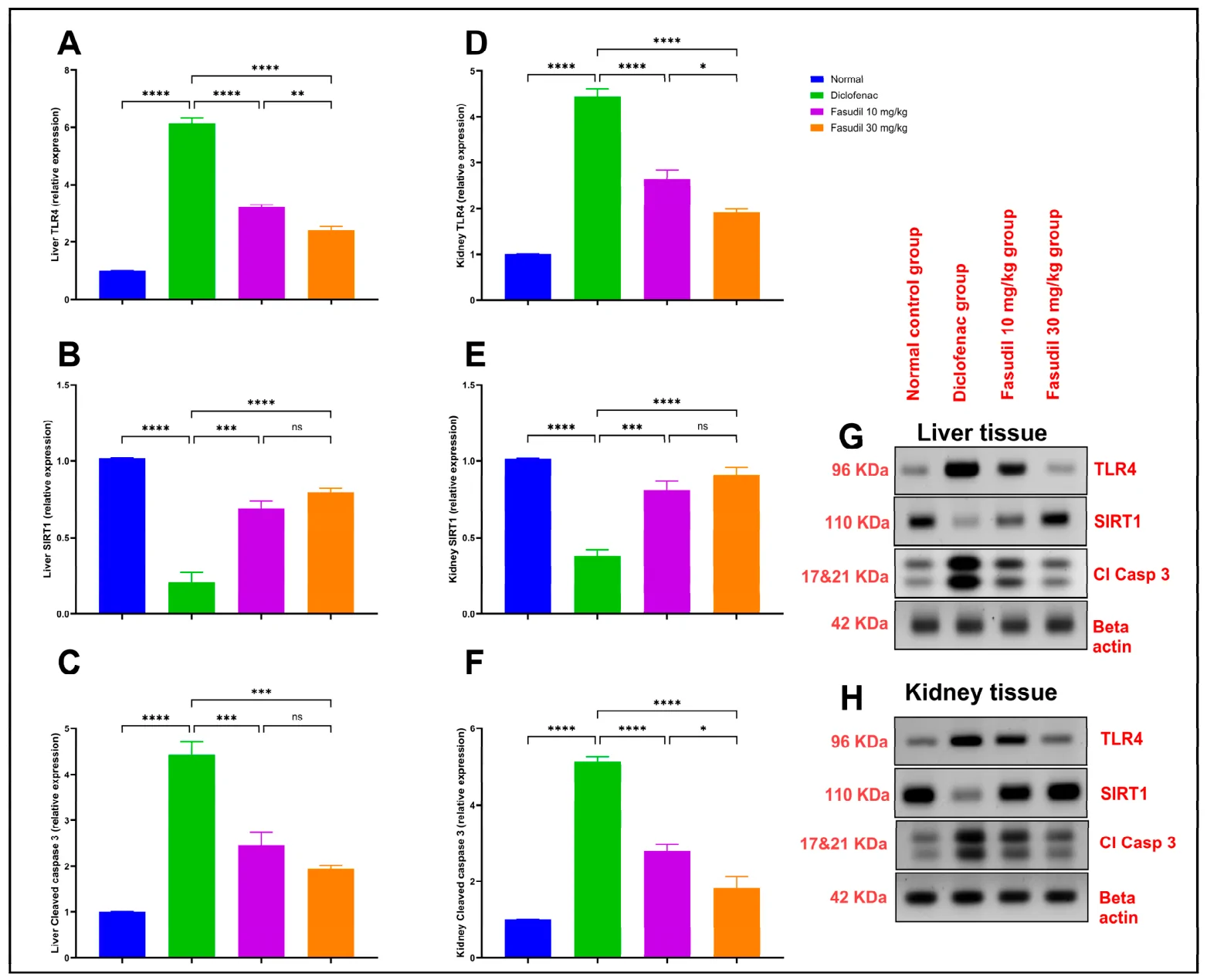
Fasudil ameliorated diclofenac-evoked hepatic and renal elevation in TLR4 expression and apoptotic injury. (**A**) TLR4 expression in liver tissue, (**B**) SIRT 1 expression in liver tissue, (**C**) cleaved caspase 3 expression in liver tissue, (**D**) TLR4 expression in kidney tissue, (**E**) SIRT 1 expression in kidney tissue, (**F**) Cleaved caspase 3 expression in kidney tissue, (**G**) Western blot images for liver tissue, (**H**) Western blot images for kidney tissue. Results are displayed as the mean ± SD (*n* = 6). Treatment conditions comprised a one-time IP challenge of diclofenac (100 mg/kg) and a 7-day oral fasudil pretreatment (10 or 20 mg/kg). A one-way ANOVA followed by Tukey’s test was used to resolve pairwise statistical differences. Asterisks define the exact significance thresholds (* *p* < 0.05, ** *p* < 0.01, *** *p* < 0.001, **** *p* < 0.0001). Original images are provided in the Supplementary Materials.

To determine if this reduction in inflammatory mediators was driven by the upstream signaling receptor, TLR4 protein expression was subsequently evaluated. The substantial systemic inflammatory response triggered by diclofenac was driven primarily by upregulation of this pathway. Diclofenac intoxication provoked a 340.2% elevation in TLR4 protein expression in the kidney and a profound 474.3% increase in TLR4 expression in the liver (both *p* < 0.0001). Fasudil pretreatment successfully abrogated this upstream inflammatory trigger across both organ systems. The 10 mg/kg dose significantly reduced TLR4 expression by 43.4% in the kidneys and 44.3% in the liver (both *p* < 0.0001). This upstream blockade was even more robust at the 30 mg/kg dose, yielding 56.7% reductions in the kidney and 58.1% in the liver compared with the diclofenac-intoxicated group (both *p* < 0.0001). Ultimately, these findings firmly demonstrate that fasudil provided potent, multi-organ protection against diclofenac toxicity by potentially modulating the upstream TLR4-driven inflammatory axis, thereby reducing the release of downstream inflammatory mediators and macrophage infiltration (Figure 6A,D).

### 3.5. Fasudil Ameliorated Diclofenac-Evoked Hepatic and Renal Apoptotic Injury via Modulation of the SIRT1/Caspase-3 Axis

Diclofenac injection markedly disrupted apoptotic homeostasis in both hepatic and renal tissues, as evidenced by a significant reduction in SIRT1 expression and a marked increase in Caspase-3 activity. In the liver tissue, SIRT1 expression was significantly decreased by about 80.3%, while caspase-3 levels exhibited a significant elevation of approximately 341.9% in comparison with the normal control group (both *p* < 0.0001). Likewise, in the kidney, SIRT1 expression decreased by 62.5%, while caspase-3 expression increased by 411.8% (both *p* < 0.0001).

In the liver, the 10 mg/kg dose of fasudil elevated SIRT1 expression by about 228.6% (*p* = 0.0002) and reduced caspase-3 expression levels by 44.5% (*p* = 0.0006), while the 30 mg/kg dose caused a more pronounced effect, increasing SIRT1 by 279.4% (*p* < 0.0001) and suppressing caspase-3 expression by about 56.2% (*p* = 0.0001) compared to the diclofenac group. In the kidney, fasudil at a dose of 10 mg/kg elevated SIRT1 expression by about 113.2% (*p* = 0.0005) and reduced caspase-3 levels by 45.5% (*p* < 0.0001), whereas the 30 mg/kg dose produced superior efficacy, elevating SIRT1 by about 140.3% and decreasing caspase-3 by 64.3% (both *p* < 0.0001). Collectively, these results demonstrated that fasudil conferred anti-apoptotic effects via restoring SIRT1-mediated survival signaling and diminishing caspase-3-dependent apoptotic activation (Figure 6B,C,E,F).

### 3.6. Fasudil Preserved Gastric Mucosal Barrier Integrity

To determine whether the suppression of local oxidative stress and inflammation successfully prevented the breakdown of the gastric epithelial barrier, a hallmark of NSAID-induced “leaky gut” and ulceration, the levels of critical TJ proteins, ZO-1, Claudin-1, and Occludin, were quantified in gastric tissue.

#### 3.6.1. ZO-1 and Claudin-1

**Figure 7:**
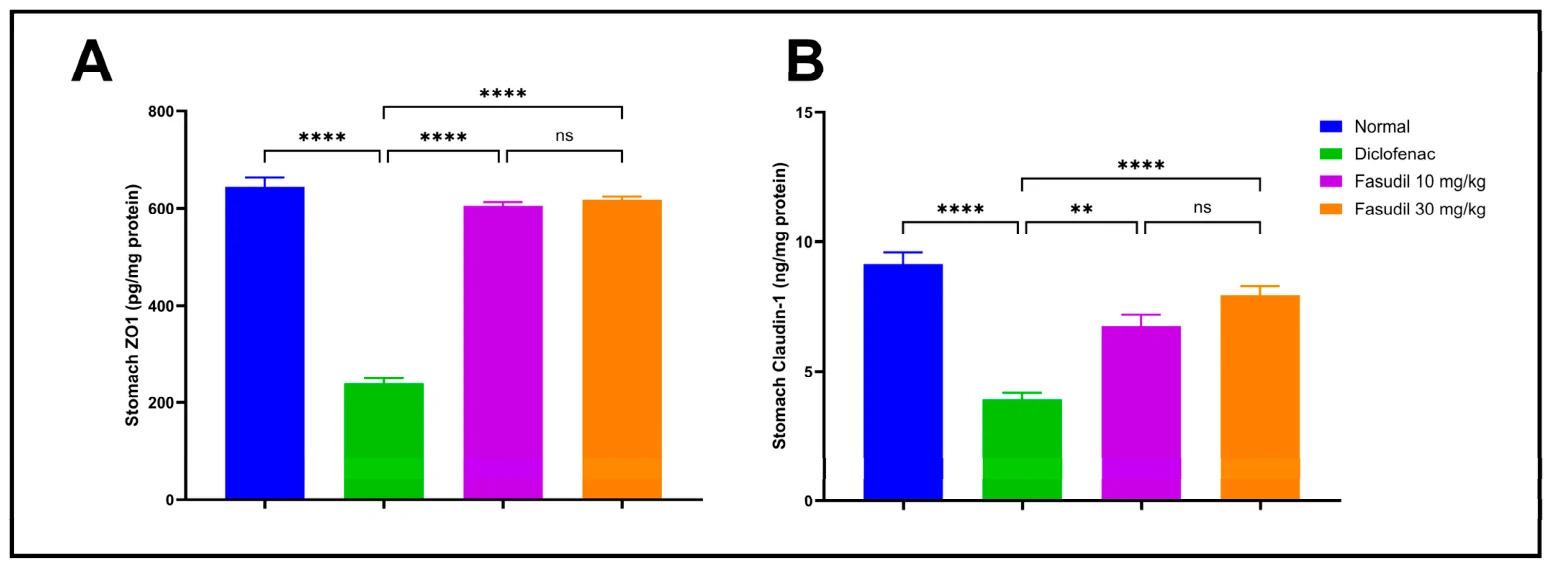
Fasudil preserved gastric barrier integrity by maintaining ZO-1 (**A**) and claudin-1 (**B**) protein expression. Quantitative values are given as the mean ± SD (*n* = 6). The in vivo treatment consisted of diclofenac (100 mg/kg, single IP injection) and fasudil (10 or 30 mg/kg, administered orally over one week). Pairwise statistical differences were calculated employing a one-way ANOVA coupled with Tukey’s test for multiple comparisons (** *p* < 0.01, **** *p* < 0.0001).

The administration of diclofenac (100 mg/kg) resulted in severe structural damage to the gastric mucosal barrier. This was evidenced by a precipitous 62.8% depletion of ZO-1 and a severe 57.4% decline in Claudin-1 relative to the normal control group (both *p* < 0.0001), indicating a substantial loss of tight junction integrity (Figure 7).

Pretreatment with fasudil effectively halted this structural collapse and preserved the mucosal barrier, demonstrating highly efficacious results. Compared to the diclofenac group, administration of fasudil at 10 mg/kg profoundly protected tight junctions from degradation, resulting in 152.3% relative preservation of ZO-1 levels (*p* < 0.0001) and an approximate 73.2% restoration of Claudin-1 levels (*p* = 0.0012). Remarkably, the protective efficacy of the 30 mg/kg fasudil dose was nearly absolute. This higher dose resulted in a 157.7% relative increase in ZO-1 levels and a 103.3% relative increase in Claudin-1 levels compared with the diclofenac-intoxicated animals (both *p* < 0.0001). Crucially, statistical comparisons revealed no significant difference in the levels of either ZO-1 (*p* = 0.4340) or Claudin-1 (*p* = 0.1769) between the 30 mg/kg fasudil group and the healthy normal controls. This indicates that the higher fasudil dose completely neutralized the diclofenac-induced damage, maintaining gastric tight junction levels at healthy, homeostatic levels.

#### 3.6.2. Occludin

Immunohistochemical analysis of occludin in gastric tissues revealed that, in the control group, stomach tissues exhibited normal structural integrity, characterized by strong positive brown staining for occludin in many glandular cells. In the diclofenac group, a severe loss of mucosal integrity was observed, with occludin severely depleted and showing only weak positive brown staining in a few glandular cells. Quantitative image analysis confirmed that the diclofenac group experienced a significant reduction in occludin expression compared to the healthy control group (*p* < 0.0001).

**Figure 8:**
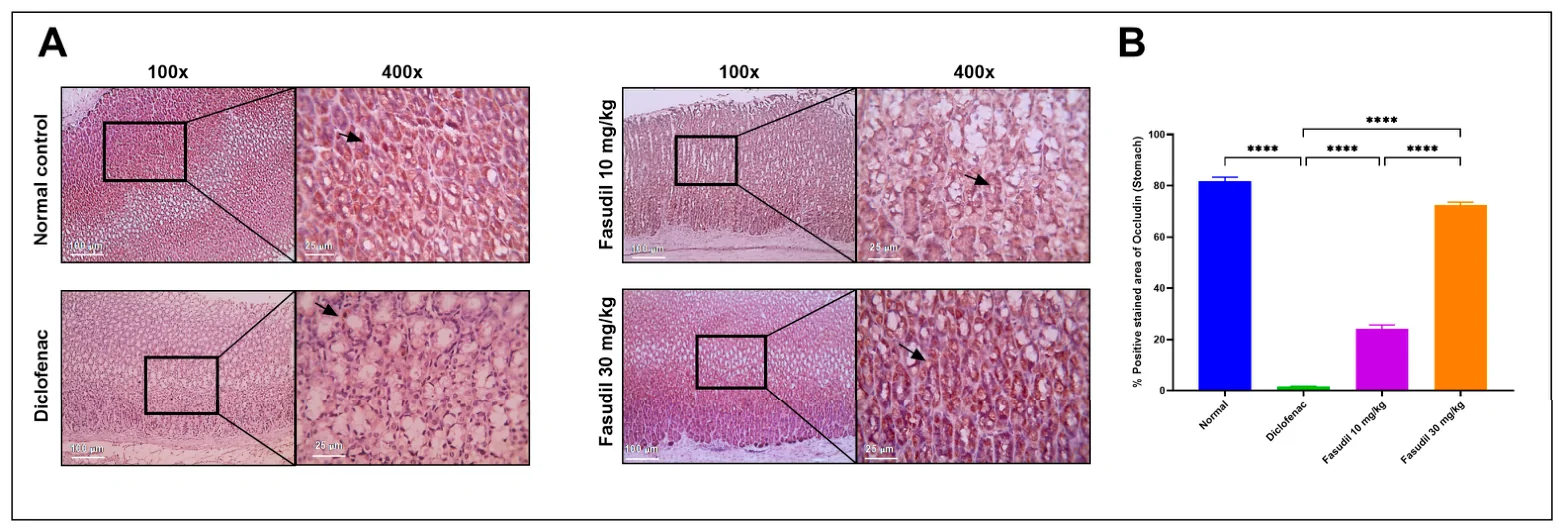
Fasudil restored gastric occludin expression. (**A**) Representative immunohistochemical images of glandular stomach sections stained for occludin. (**B**) Occludin immunostaining histogram. Black arrows: positive brown staining in a few glandular cells. Mayer’s hematoxylin was utilized as the nuclear counterstain for the immunohistochemical preparations. Microscopic evaluations were conducted at 100× (scale = 100 μm) and 400× (scale = 25 μm). All quantitative values are reported as the mean ± SD for cohorts of six animals. The in vivo model consisted of a seven-day intragastric fasudil pretreatment (10 or 30 mg/kg), which was followed by a single 100 mg/kg IP diclofenac injection on day 8. Group comparisons were resolved utilizing a one-way ANOVA alongside Tukey’s post hoc correction (**** *p* < 0.0001). Original images are provided in the Supplementary Materials.

Treatment with 10 mg/kg fasudil visibly increased positive brown staining in some glandular cells, significantly increasing occludin expression by 1511.3% relative to the diclofenac group (*p* < 0.0001). Notably, 30 mg/kg Fasudil exerted a profound restorative effect, resulting in strong positive brown staining in many glandular cells. Quantitative evaluation revealed that 30 mg/kg fasudil markedly improved structural integrity, increasing occludin expression by 4733.3% compared to the diclofenac group (*p* < 0.0001). Interestingly, there was a significant increase in occludin expression level in the 30 mg/kg fasudil group compared to the 10 mg/kg fasudil group (*p* < 0.0001) (Figure 8).

### 3.7. Fasudil Ameliorated Histopathological Alterations in the Liver, Kidney, and Stomach Tissues

#### 3.7.1. Hepatic Tissues

**Figure 9:**
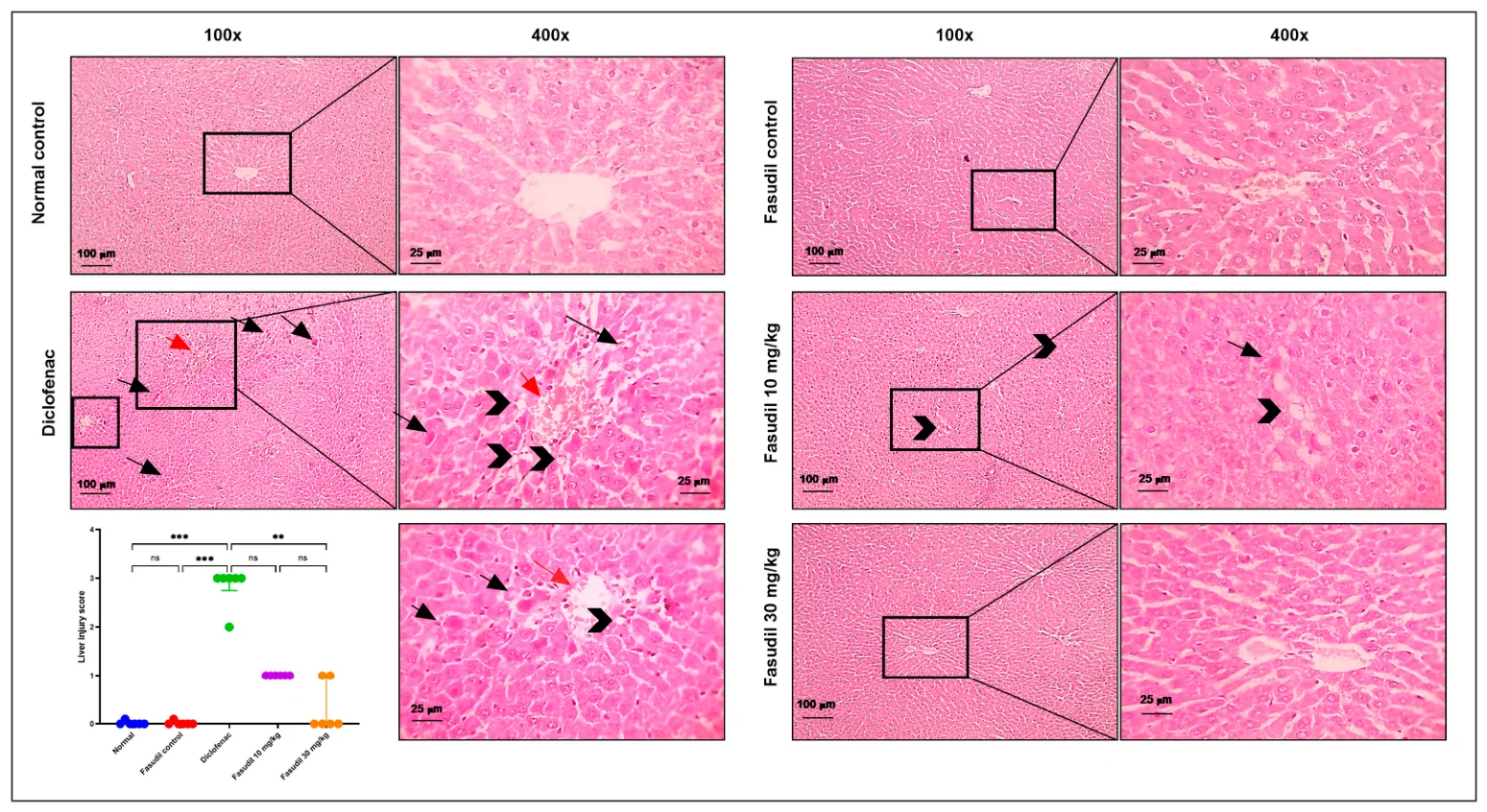
Fasudil reverses diclofenac-induced morphological damage in hepatic tissue. Representative H&E micrographs from the normal and fasudil-only control cohorts display an intact hepatic architecture, characterized by healthy hepatocytes, well-preserved portal tracts, and normal central veins and sinusoids. Conversely, the diclofenac-exposed tissues exhibit pronounced architectural injury, highlighted by central vein congestion (red arrows), notable cytoplasmic vacuolization (black arrowhead), and numerous condensed, apoptotic hepatocytes presenting with intensely eosinophilic cytoplasm (thin black arrow). Treatment with 10 mg/kg fasudil noticeably mitigated these pathologies, revealing only occasional apoptotic cells (thin black arrow) and mild vacuolation (black arrowhead). Administration of the 30 mg/kg fasudil dose facilitated a near-complete structural recovery, with the hepatic parenchyma, portal areas, and central veins appearing essentially normal. Representative micrographs are provided at magnifications of 100× (100 μm scale) and 400× (25 μm scale). Hepatic damage is visualized using scatter dot plots, with quantitative results reported as the median and interquartile range (IQR) for cohorts of six animals. The in vivo protocol involved an intragastric pretreatment with fasudil (10 or 30 mg/kg) over seven days, culminating in a single intraperitoneal diclofenac injection (100 mg/kg) on day eight. Variance among groups was evaluated via a Kruskal–Wallis analysis followed by Dunn’s post hoc method, where ** indicates *p* < 0.01 and *** indicates *p* < 0.001. Original images are provided in the Supplementary Materials.

In the liver, both the normal control and fasudil control groups exhibited normal architecture, featuring intact hepatocytes, central veins, portal areas, and sinusoids. Conversely, the diclofenac group displayed severe hepatic damage, characterized by many shrunken apoptotic hepatocytes with strongly eosinophilic cytoplasm, cytoplasmic vacuolation in surrounding cells, and congested central veins. This damage was reflected by a pronounced increase in the histopathological hepatic injury score (*p* = 0.0009). Treatment with 10 mg/kg fasudil mitigated this damage, reducing the hepatic injury score (*p* < 0.9999), leaving few shrunken apoptotic hepatocytes and some mild cytoplasmic vacuoles. Remarkably, 30 mg/kg fasudil restored hepatic architecture to near-normal levels, significantly reduced the injury score (*p* = 0.0099), and returned it to levels not significantly different from those of the healthy control groups (*p* < 0.9999) (Figure 9).

#### 3.7.2. Renal Tissues

**Figure 10:**
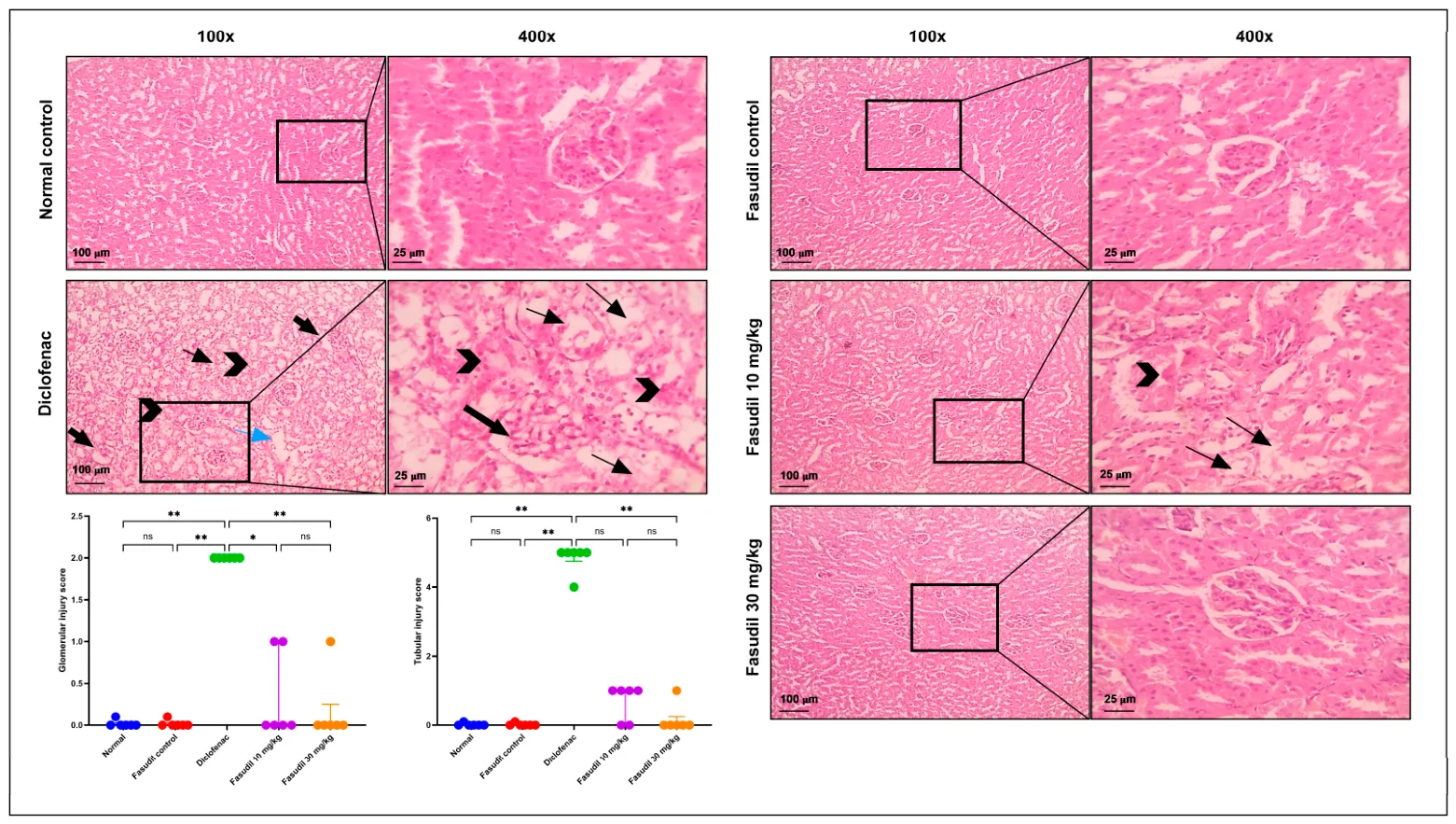
Attenuation of diclofenac-triggered nephrotoxicity by fasudil. Histological evaluation of H&E-stained renal sections confirmed unblemished baseline structures—including normal glomeruli, tubules, and interstitial spaces—across both control groups. Diclofenac exposure induced extensive tissue deterioration, marked by severe diffuse hydropic degeneration and pyknotic nuclei in the epithelial lining (thin black arrow), focal coagulative necrosis (black arrowhead), retraction of the glomerular tuft alongside an expanded capsular space (thick black arrow), and visible interstitial edema (blue arrows). The 10 mg/kg fasudil intervention notably diminished these pathologies, leaving only minor hydropic tubular swelling (thin black arrow) and occasional necrotic cells (black arrowhead). Furthermore, the higher fasudil dosage (30 mg/kg) resulted in a near-total preservation of the renal microanatomy, closely resembling the healthy control morphology. Semi-quantitative assessments of glomerular and tubular injury are graphed as scatter dot plots, detailing the median and IQR (*n* = 6). Representative tissue sections are visualized at 100× (scale = 100 μm) and 400× (scale = 25 μm) magnifications. For the in vivo model, daily intragastric fasudil (10 or 30 mg/kg) was given for a week prior to a solitary 100 mg/kg IP challenge of diclofenac on the eighth day. Group differences were evaluated employing a Kruskal–Wallis test coupled with Dunn’s multiple comparisons method (* *p* < 0.05, ** *p* < 0.01). Original images are provided in the Supplementary Materials.

Similar protective effects were observed in the renal tissues (Figure 10). Kidneys from the normal control and fasudil control groups showed normal glomeruli, tubules, and interstitial tissue. The diclofenac group showed severe diffuse hydropic degeneration with pyknotic nuclei and coagulative necrosis in the tubular epithelium, along with shrunken glomerular tufts, dilated Bowman’s spaces, and interstitial edema. This resulted in profound elevations in both tubular (*p* = 0.0022) and glomerular (*p* = 0.0031) injury scores. Treatment with 10 mg/kg fasudil provided substantial protection, effectively reversing glomerular and tubular damage to mild hydropic degeneration and focal coagulative necrosis. The 30 mg/kg fasudil dose further enhanced this protection, significantly reduced the tubular (*p* = 0.0035) and glomerular (*p* = 0.0044) injury score, and preserved normal glomerular and tubular morphology.

#### 3.7.3. Gastric Tissue

**Figure 11:**
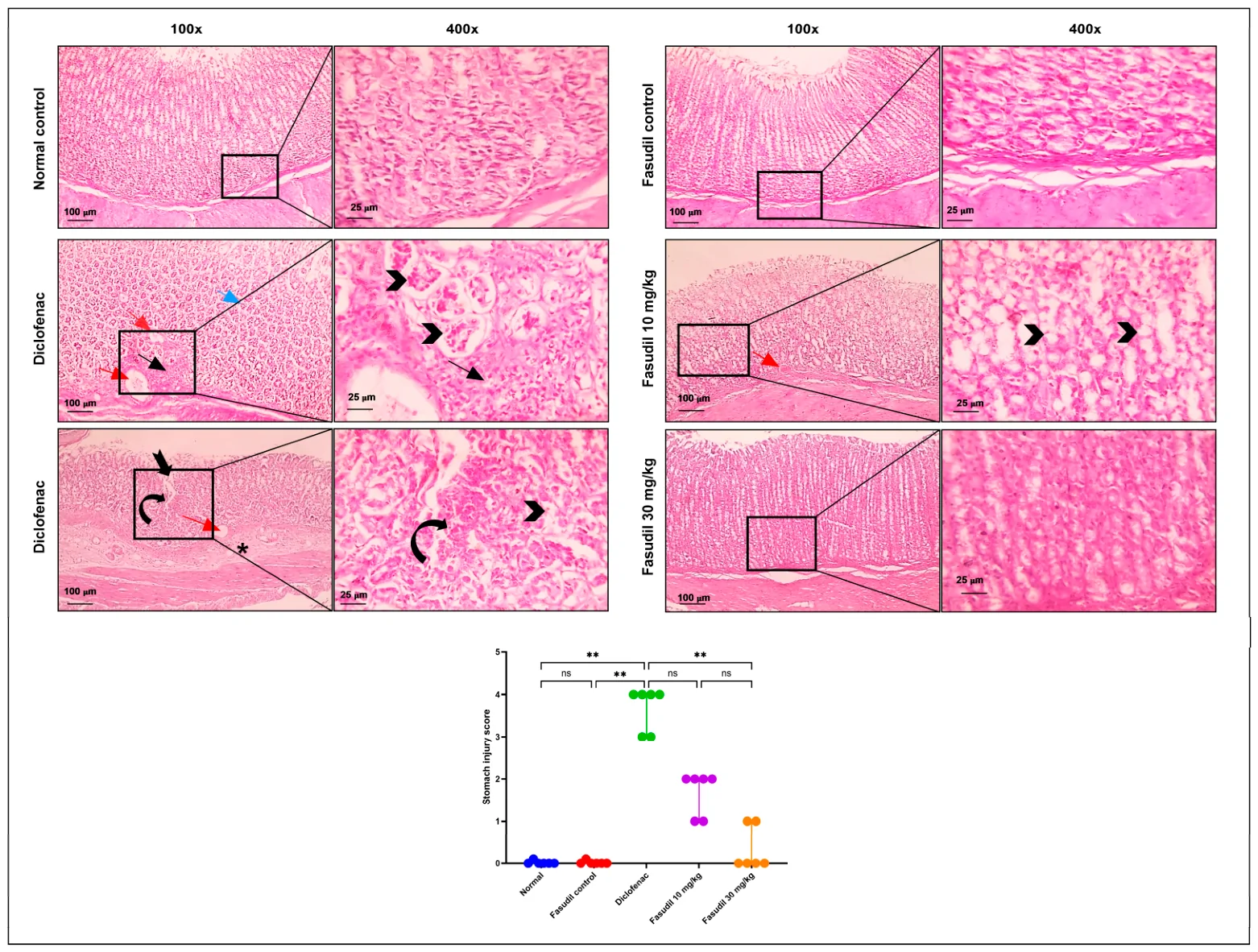
Attenuation of diclofenac-triggered gastric histopathology by fasudil. Microscopic evaluation of H&E-stained stomach sections confirmed unblemished baseline structures—including normal glandular mucosa and submucosa—in both control groups. Diclofenac exposure induced severe tissue disruption, marked by atrophic glandular contraction (black arrowhead), interstitial edema (blue arrow), fibrotic changes (thin black arrow), and vascular dilation (red arrows). Further evaluations of diclofenac-treated specimens demonstrated focal necrotizing mucosal lesions (curved black arrow) and ulcer formation (thick black arrow), alongside prominent submucosal fibrosis, leukocyte recruitment (*), and enlarged capillaries (red arrows). The 10 mg/kg fasudil intervention notably diminished these severe outcomes, presenting only mild vacuolar degeneration of the glands (black arrowheads) and slight mucosal vessel dilation (red arrows), with an intact submucosal layer. Furthermore, the higher fasudil dosage (30 mg/kg) resulted in a near-total preservation of the gastric microanatomy, closely resembling healthy control morphology. Stomach pathology scores are plotted as medians with their IQR (*n* = 6) using scatter dot plots. Microscopic evaluation was performed at 100× (100 μm bar) and 400× (25 μm bar). The in vivo phase consisted of intragastric fasudil (10 or 30 mg/kg) for 7 days, prior to a singular diclofenac IP dose (100 mg/kg) on day 8. Variance was resolved utilizing the Kruskal–Wallis method coupled with Dunn’s multiple comparisons test (** *p* < 0.01). Original images are provided in the Supplementary Materials.

Finally, we evaluated the glandular stomach mucosa and submucosa (Figure 11). While control tissues (normal control and fasudil control) were entirely normal, the diclofenac group exhibited severe gastric injury, including shrunken atrophied glandular structures, marked interstitial edema, mucosal coagulative necrosis, ulceration, dilated blood vessels, severe submucosal fibrosis, and leukocytic infiltration. Consequently, the stomach injury score elevated significantly (*p* = 0010). Treatment with 10 mg/kg fasudil limited damage to mild vacuolar degeneration in the glandular epithelium and mildly dilated mucosal blood vessels, resulting in a lower injury score. The 30 mg/kg fasudil dose exerted near-complete mucosal rescue, preventing ulceration and fibrosis, reducing the histopathological score (*p* = 0.0067), and maintaining normal mucosa and submucosa.

## 4. Discussion

While diclofenac remains one of the most globally consumed NSAIDs for pain and inflammation management, its clinical utility is severely hampered by its propensity to induce idiosyncratic drug-induced liver injury (DILI), acute kidney injury (AKI), and pronounced gastric ulcerations. Previous research has largely addressed these organ toxicities in isolation or targeted downstream symptoms rather than upstream mechanisms [2,3,4,5]. Historically, the diverse mechanisms driving these diclofenac-induced multi-organ toxicities have been attributed to ROS accumulation, mitochondrial dysfunction, and sterile inflammation [6,7,17]. Recent peer-reviewed studies increasingly emphasize the role of the innate immune response, noting that diclofenac toxicity heavily relies on the activation of TLR4, which drives downstream apoptotic cell death across tissues [9,50,51]. However, previous research has largely addressed these organ toxicities in isolation or targeted downstream, end-stage inflammatory markers rather than identifying a unifying upstream pharmacological target.

The novelty of the current study lies in its comprehensive, multi-organ approach to this global health challenge. To our knowledge, this is the first study to demonstrate that pharmacological inhibition of the ROCK2-associated pathway using fasudil can concurrently and dose-dependently abrogate diclofenac-induced hepatotoxicity, nephrotoxicity, and gastric mucosal barrier breakdown. ROCK2, a crucial downstream effector of RhoA, is deeply implicated in exacerbating oxidative stress, driving pro-inflammatory signaling cascades, and facilitating actin-driven degradation of epithelial TJ proteins, including ZO-1 and Claudins [26]. Recent investigations into fasudil have validated its potent anti-inflammatory and cytoprotective properties in isolated liver fibrosis models and septic kidney injury models [52]. Building upon this, by systematically tracking the ROCK2/TLR4/SIRT1 molecular axis alongside structural TJ integrity in our in vivo model, we have mapped a unified molecular pathology for NSAID-induced tissue destruction. Specifically, our findings establish that fasudil effectively dismantles this pathological cascade across multiple organ systems by acting as a master upstream regulator—suppressing TLR4-driven inflammation, preventing mucosal barrier collapse, and restoring SIRT1-mediated cellular survival.

The administration of diclofenac triggered catastrophic organ failure in our model, characterized by profound elevations in serum transaminases (AST, ALT), substantial increase in BUN and creatinine, and severely compromised urinary creatinine clearance. This functional collapse was tightly coupled with an overwhelming oxidative imbalance, evidenced by depleted GSH and TAC levels alongside elevated MDA levels in the liver, kidneys, and stomach. These findings strongly corroborate earlier studies [6,7,53], which established that the metabolic activation of diclofenac generates ROS that directly induce lipid peroxidation and subsequent hepatorenal necrosis. Remarkably, fasudil pretreatment—particularly at the 30 mg/kg dose—fully restored hepatorenal function and neutralized the oxidative burden. This suggests that ROCK2 overactivation is not merely a byproduct of diclofenac toxicity but a crucial driver of oxidative stress. By inhibiting ROCK2, fasudil likely prevents the cytoskeletal derangements that exacerbate mitochondrial oxidative injury, thereby preserving endogenous antioxidant reservoirs and maintaining cellular homeostasis.

A core finding of our study is the precise mapping of the “sterile inflammation” cascade initiated by diclofenac toxicity. Unlike pathogen-driven inflammation, NSAID-induced organ injury generates Damage-Associated Molecular Patterns (DAMPs) from stressed or dying cells [54]. Our data reveal that diclofenac toxicity induced a marked systemic overactivation of ROCK2, which acted as an indispensable upstream catalyst for TLR4 upregulation. Mechanistically, ROCK2 is not merely a signaling kinase; it heavily regulates cytoskeletal dynamics, which are required for the proper surface clustering, stabilization, and activation of innate immune receptors like TLR4 [55]. This receptor overactivation subsequently unleashed a severe inflammatory storm, driving the robust downstream tissue expression of NF-κB, IL-1β, and TNF-α [56].

The consequences of this TLR4-driven cascade are highly destructive. Upon activation, TLR4 facilitates the nuclear translocation of the transcription factor NF-κB, which in turn regulates the expression of a vast array of pro-inflammatory genes [57]. This results in the substantial localized release of TNF-α—a potent mediator of tissue necrosis and apoptosis—and IL-1β, which further amplifies inflammasome activation and drives pyroptotic cell death [58]. Furthermore, this localized cytokine storm acts as a chemoattractant, stimulating the significant infiltration of CD68-positive macrophages [59]. Once recruited to the hepatic and renal microenvironments, these macrophages typically polarize into an aggressive M1 phenotype, secreting additional cytokines and creating a catastrophic positive feedback loop that accelerates irreversible organ necrosis [60].

Our findings position fasudil as a highly potent anti-inflammatory agent that works by severing this ROCK2-TLR4 connection at its root, rather than merely mitigating downstream symptoms. By inhibiting ROCK2, fasudil likely disrupts the cytoskeletal scaffolding required for TLR4 signaling, effectively slashing TLR4 tissue expression. Consequently, this upstream blockade starves the NF-κB pathway of its primary trigger, reducing downstream effectors such as IL-1β and TNF-α to near-baseline levels and completely halting the recruitment of CD68+ macrophages.

This mechanism aligns beautifully with the broader literature and positions ROCK inhibition as a superior therapeutic strategy to targeting individual cytokines. For instance, recent work demonstrates that fasudil significantly improves inflammatory conditions like neutrophilic asthma by actively modulating macrophage polarization and suppressing the NF-κB/TLR pathway [24]. Similarly, other contemporary studies highlight TLR4 as the central molecular node in driving sterile tissue inflammation across various models of drug-induced and ischemic organ injury [25]. Ultimately, our data definitively show that ROCK2 is an indispensable upstream regulator of NSAID-induced innate immune activation, and its targeted pharmacological inhibition effectively short-circuits the inflammatory feedback loop before irreversible tissue damage occurs.

Beyond initiating inflammation, diclofenac toxicity critically dictates cellular fate by driving profound pro-apoptotic and pyroptotic actions, which ultimately culminate in widespread hepatocyte and tubular necrosis [17,18]. In our study, this transition to programmed cell death was manifested by a substantial upregulation in cleaved caspase-3—the primary executioner protease responsible for systematically dismantling cellular architecture and driving apoptosis [61]. Crucially, this execution phase was accompanied by a simultaneous, severe suppression of SIRT1, an NAD+-dependent histone deacetylase that serves as a master metabolic regulator of cellular survival and stress resistance [62]. The ability of fasudil to dose-dependently rescue these tissues represents a vital, multi-pronged therapeutic mechanism. Fasudil does not merely suppress the initial damage; it actively promotes tissue resilience by upregulating SIRT1 expression while concurrently silencing the upstream TLR4 inflammatory axis. SIRT1 is highly regarded for its profound anti-apoptotic and antioxidant capabilities, which it achieves by deacetylating key downstream regulatory proteins (such as p53, FOXO, and NF-κB) to prevent premature cell death and bolster endogenous antioxidant defenses [63].

The robust restoration of SIRT1 by fasudil directly counters the pathological mechanisms detailed in recent literature [9,10]. These studies illustrate how unmitigated oxidative stress and TLR4-driven inflammatory signals overwhelm and heavily suppress cellular survival networks. When SIRT1 is depleted by diclofenac toxicity, the inhibitory “brakes” on the highly inflammatory TXNIP/NLRP3 inflammasome axis are released. Free from SIRT1’s regulation, TXNIP activates the NLRP3 inflammasome, triggering pyroptosis (a highly inflammatory form of lytic cell death) and further exacerbating tissue injury.

By restoring SIRT1 levels, fasudil effectively short-circuits this destructive cycle. The preserved SIRT1 activity suppresses TXNIP, thereby keeping the NLRP3 inflammasome inactive and preventing pyroptosis [64]. Simultaneously, the upstream blockade of ROCK/TLR4 signaling halts the cleavage and activation of Caspase-3, directly stopping the apoptotic execution cascade. Ultimately, by actively promoting SIRT1-mediated survival networks and halting Caspase-3 cleavage, fasudil fundamentally shifts the cellular microenvironment from a terminal execution phase back toward a state of recovery and homeostatic repair [65].

Perhaps the most clinically relevant limitation of NSAID therapy is the destruction of the gastric mucosal barrier, often termed “leaky gut,” leading to fatal ulcerations [12,13,19]. In our model, diclofenac completely decimated the structural integrity of the stomach, obliterating the expression of essential TJ proteins, including ZO-1, Claudin-1, and Occludin.

ROCK signaling physically facilitates the actin-driven degradation and internalization of these crucial TJ proteins. Active comparison with existing studies confirms that overactive RhoA/ROCK signaling destabilizes the actomyosin ring, pulling TJs apart [26]. Our immunohistochemical and biochemical analyses reveal that the 30 mg/kg dose of fasudil exerted a near-absolute restorative effect, preventing TJ depletion and mucosal ulceration. This provides conclusive evidence that targeting the ROCK pathway is a highly effective strategy for preventing NSAID-induced enteropathy, acting precisely at the structural level to maintain barrier impermeability.

### Limitations

This study has some limitations. First, it utilized an acute, high-dose intraperitoneal diclofenac model, which does not fully replicate the chronic, low-dose oral exposure typically seen in clinical NSAID toxicity. Therefore, further studies using clinically relevant diclofenac dosing regimens and chronic exposure models are important to validate the safe applicability of the present findings. Second, pharmacokinetic analysis of fasudil was not performed in the present study; So, the possible relations between drug exposure and the observed effects could not be established. Third, while fasudil is a potent pharmacological ROCK inhibitor, potential off-target effects necessitate future validation of the ROCK2/TLR4 axis using genetic knockout models. Fourth, in the current study, although fasudil is a nonselective ROCK inhibitor, the activity or expression of ROCK1 was not evaluated. Therefore, the possible contribution of ROCK1 to the observed protective effects of fasudil cannot be entirely ruled out. Investigation of the individual role of ROCK1 and ROCK2 in diclofenac-induced organ injury is planned to be part of our future research scope. Fifth, the exclusive use of male rats precludes assessing potential sexual dimorphism in drug metabolism and inflammatory responses. Sixth, although mitochondrial injury was suggested by the observed molecular and biochemical alterations, direct evaluation of mitochondrial activity was not performed. Future studies evaluating mitochondrial biogenetics and respiratory functions are needed to better clarify the contribution of mitochondrial dysfunction in diclofenac-evoked organ injury and its modulation by fasudil. Seventh, although key tight junction proteins were assessed, future studies including additional barrier integrity markers, such as claudin-18 and mucins expression, and direct functional permeability assays, would strengthen our findings regarding preservation of gastric mucosal barrier function. Finally, because this study employed a prophylactic pretreatment design, it remains unknown whether fasudil can therapeutically reverse established diclofenac-induced multi-organ damage.

## 5. Conclusions

In summary, diclofenac-induced multi-organ toxicity is driven by an aggressive interplay of ROCK2/TLR4-mediated inflammation, substantial oxidative stress, suppression of SIRT1 survival pathways, and the physical degradation of tight junctions. Fasudil acts as a master regulator, intervening far upstream to silence ROCK2. This pharmacological blockade offers profound, dose-dependent protection across the liver, kidneys, and gastric mucosa, highlighting the RhoA/ROCK axis as a premier therapeutic target for mitigating the severe adverse effects of chronic NSAID administration.

## Data Availability

The data presented in this study are available on reasonable request from the corresponding author due to ongoing research associated with the dataset.

## Supplementary Materials

The following supporting information can be downloaded at: https://www.mdpi.com/article/10.3390/jox16040144/s1, FileS1: Original images of Figure 4, Figure 5, Figure 6, Figure 8, Figure 9, Figure 10 and Figure 11.
