# Supplementary material for: Fasudil Protects Against Diclofenac-Induced Hepatorenal and Gastric Injury via Modulation of ROCK/TLR4/SIRT1 Signaling and Preservation of Tight Junctions
Source: J Xenobiot. 2026 Aug 2;16(4):144. doi: 10.3390/jox16040144 (PMC13514739; doi:10.3390/jox16040144)
Supplement: Supplementary file 1 [file jox-16-00144-s001.zip › jox-4446506-supplementary.pdf]

Supplementary Materials: Fasudil Protects Against Diclofenac-Induced Hepatorenal and Gastric Injury via Modulation of ROCK/TLR4/SIRT1 Signaling and Preservation of Tight Junctions

Ali H. Al-Baldawi, Mahmoud M. Samaha and Marwa S. Zaghloul

Original images of Figures 4, 5, 6, 8, 9, 10, and 11

Figure 4A

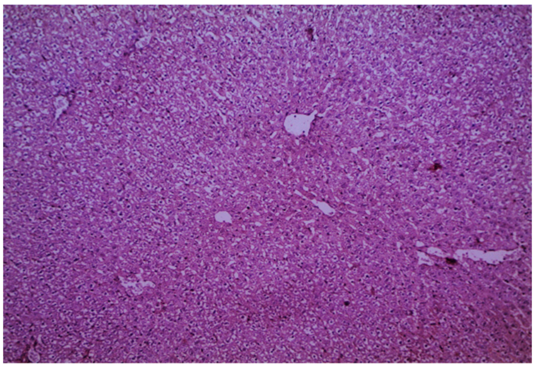

Normal control X100

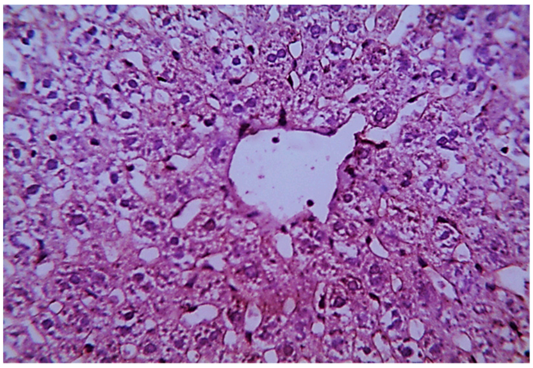

Normal control X400

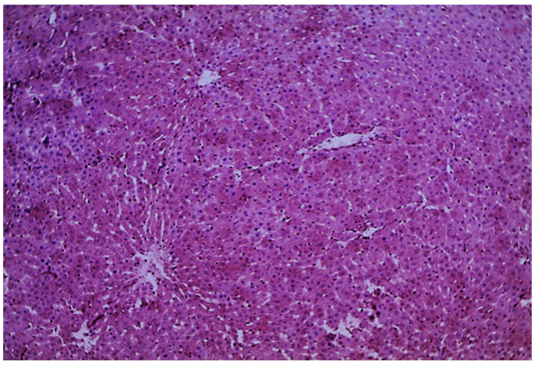

Diclofenac group X100

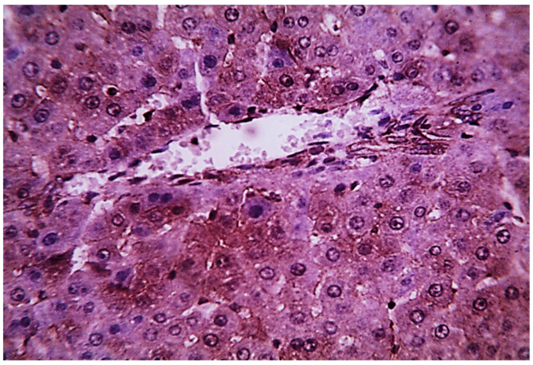

Diclofenac group X400

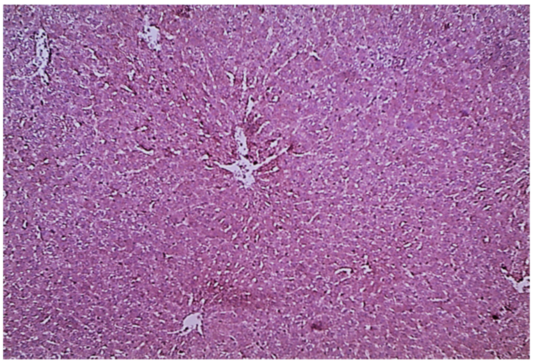

Fasudil 10 mg/kg group X100

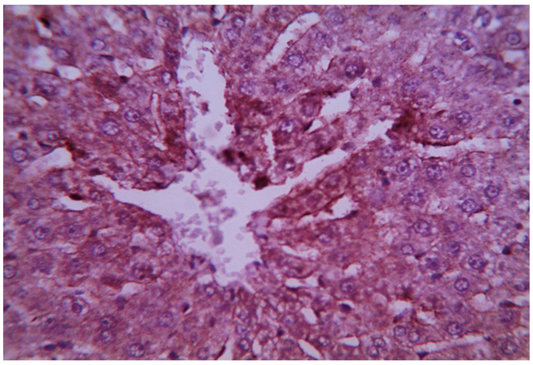

Fasudil 10 mg/kg group X400

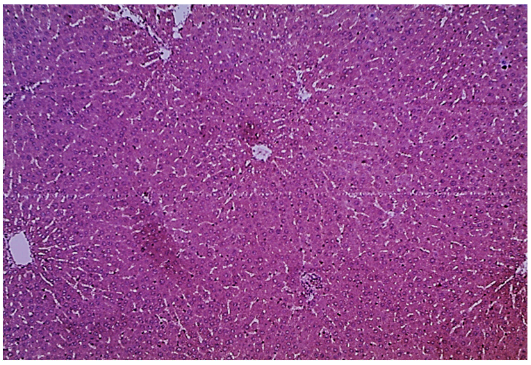

Fasudil 30 mg/kg group X100

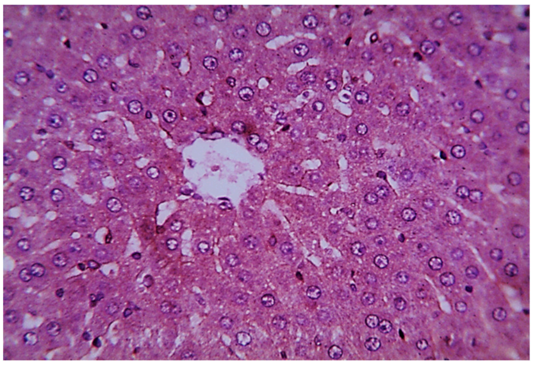

Fasudil 30 mg/kg X400

Figure 4B

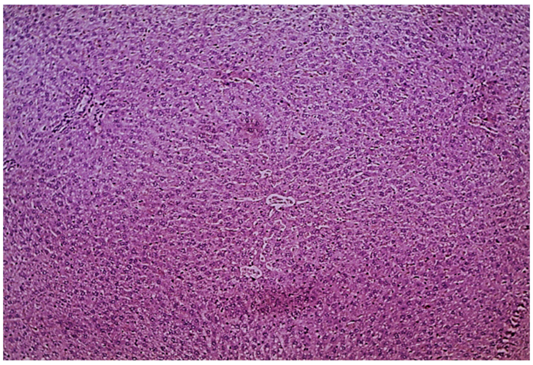

Normal control X100

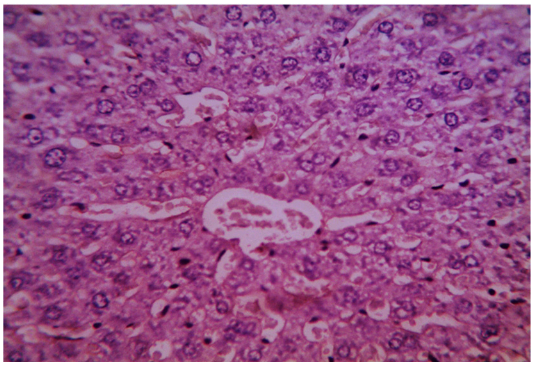

Normal control X400

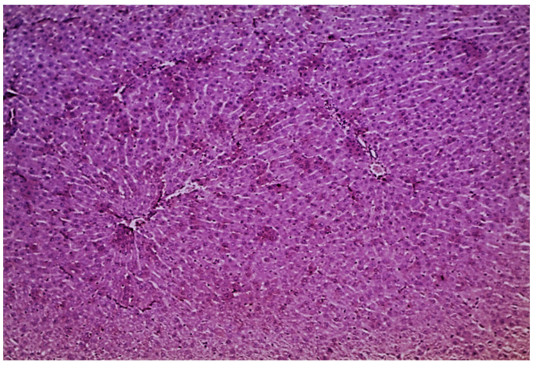

Diclofenac group X100

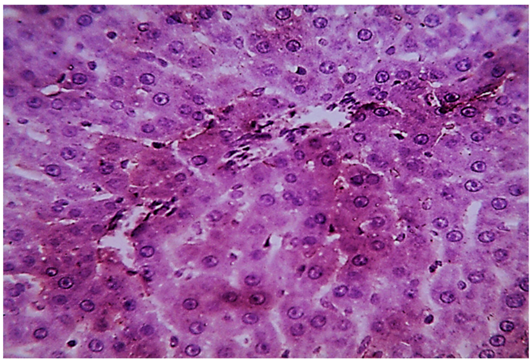

Diclofenac group X400

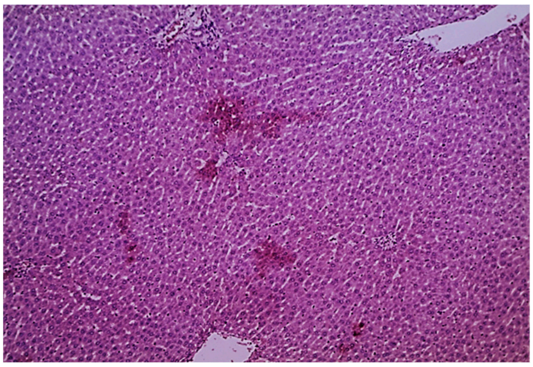

Fasudil 10 mg/kg group X100

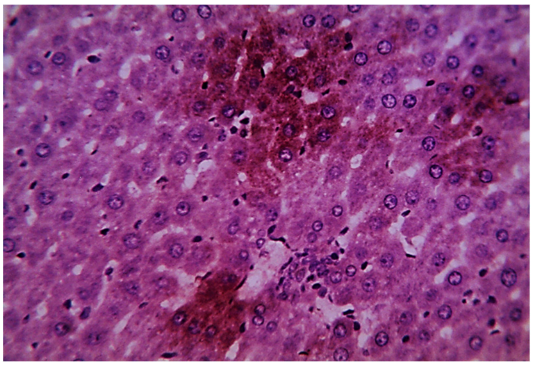

Fasudil 10 mg/kg group X400

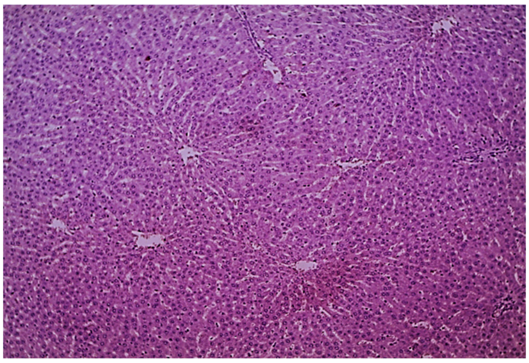

Fasudil 30 mg/kg group X100

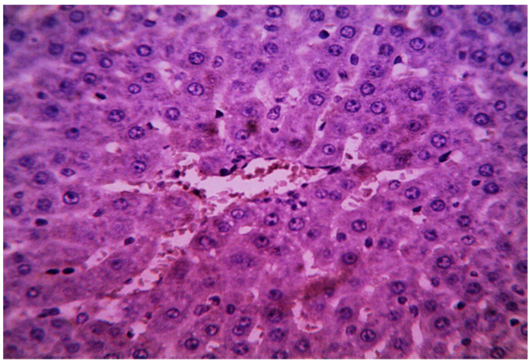

Fasudil 30 mg/kg group X400

Figure 5A

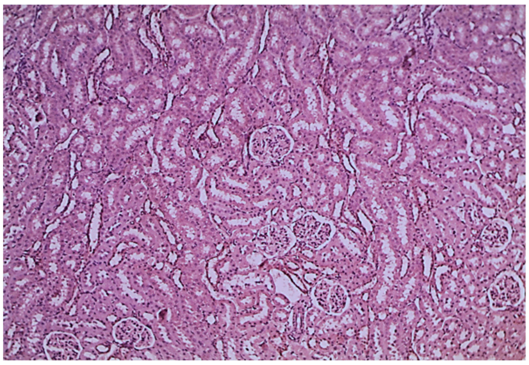

Normal control X100

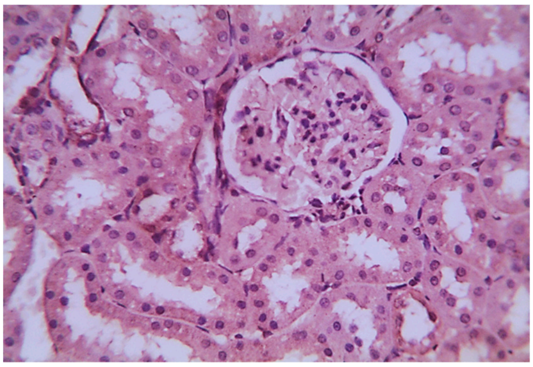

Normal control X400

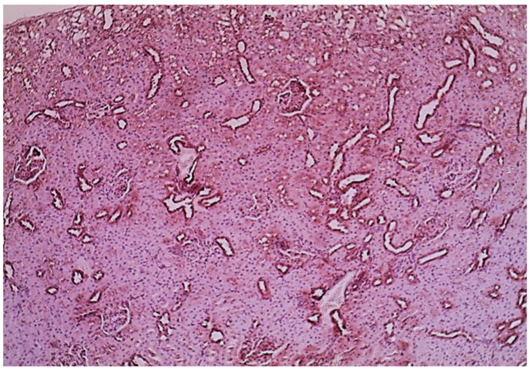

Diclofenac group X100

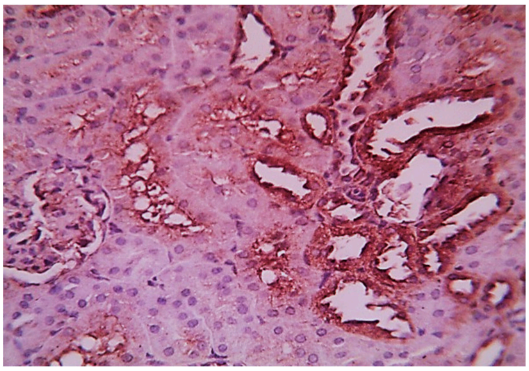

Diuclofenac group X400

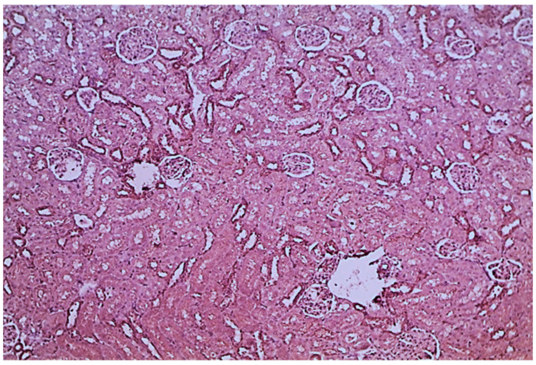

Fasudil 10 mg/kg group X100

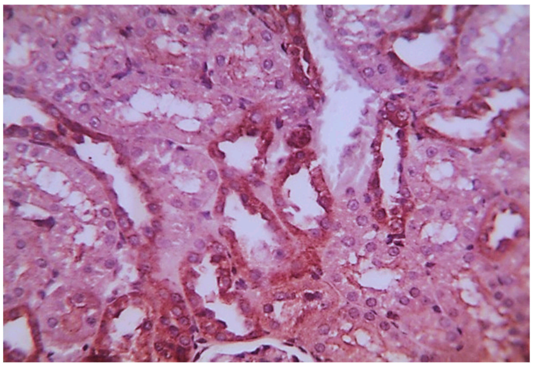

Fasudil 10 mg/kg group X400

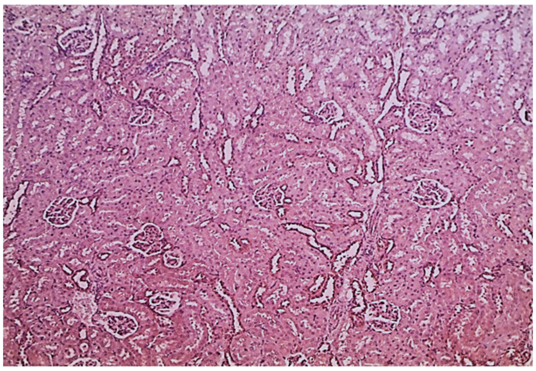

Fasudil 30 mg/kg group X100

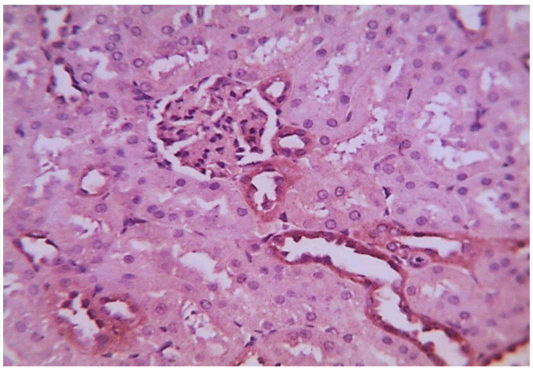

Fasudil 30 mg/kg group X400

Figure 5B

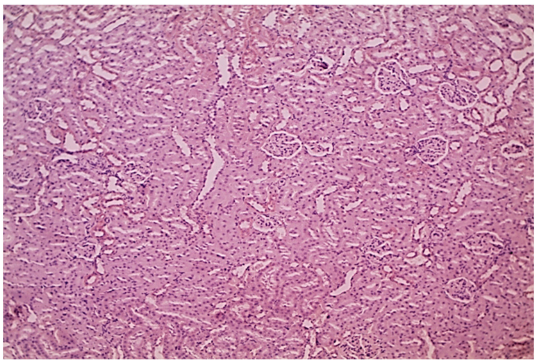

Normal control X100

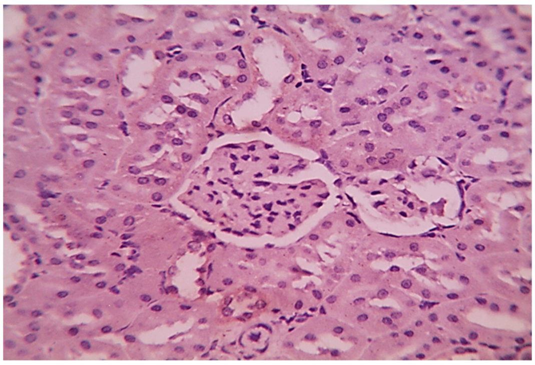

Normal control X400

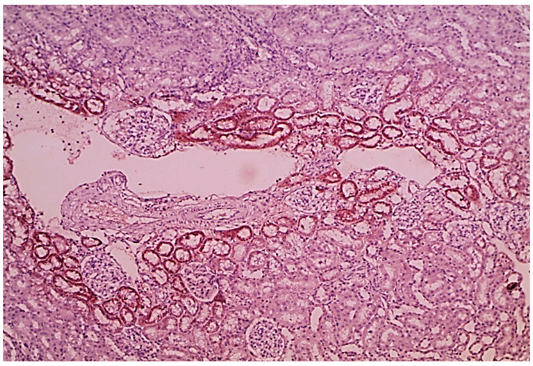

Diclofenac group X100

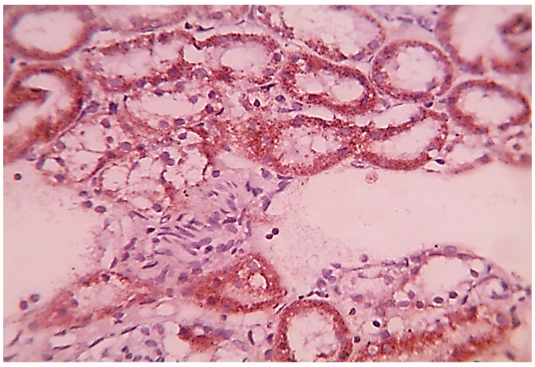

Diclofenac group X400

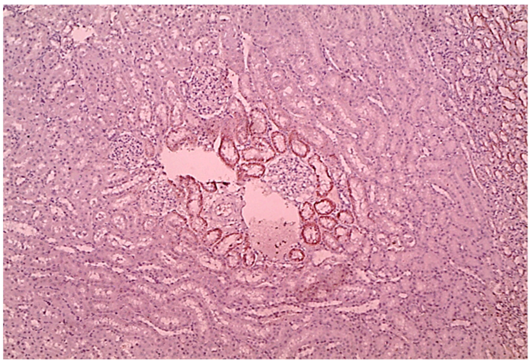

Fasudil 10 mg/kg group X100

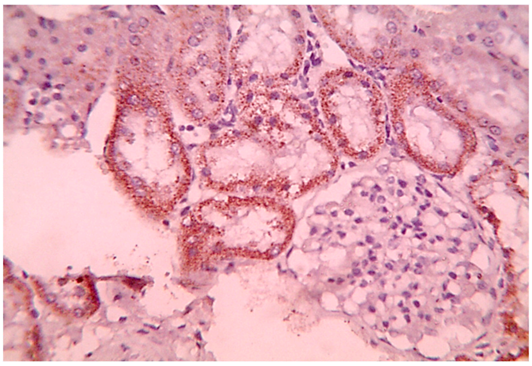

Fasudil 10 mg/kg X400

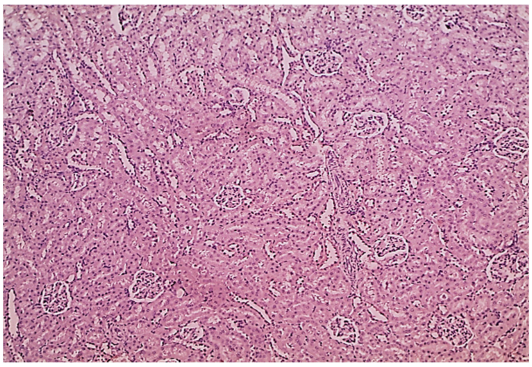

Fasudil 30 mg/kg group X100

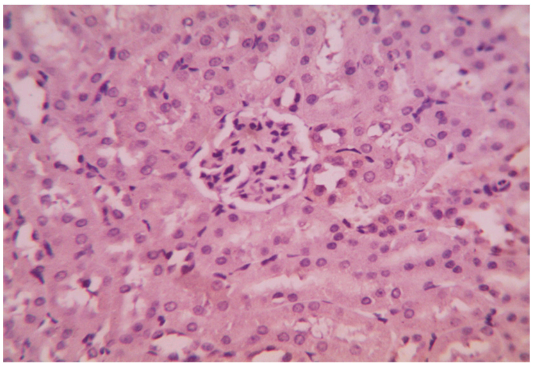

Fasudil 30 mg/kg group X400

Figure 6 G/H

β actin Kidney

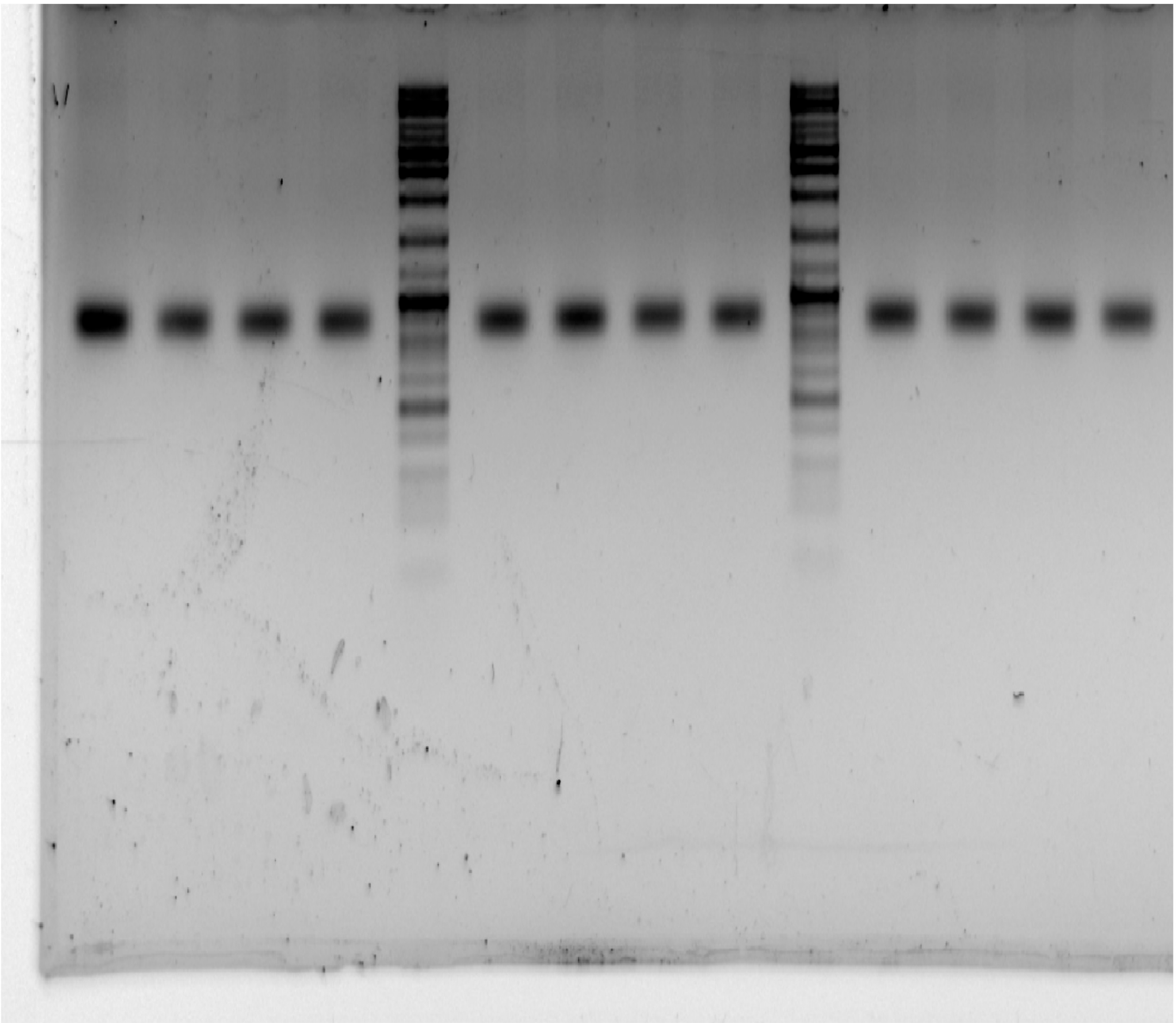

β actin Liver

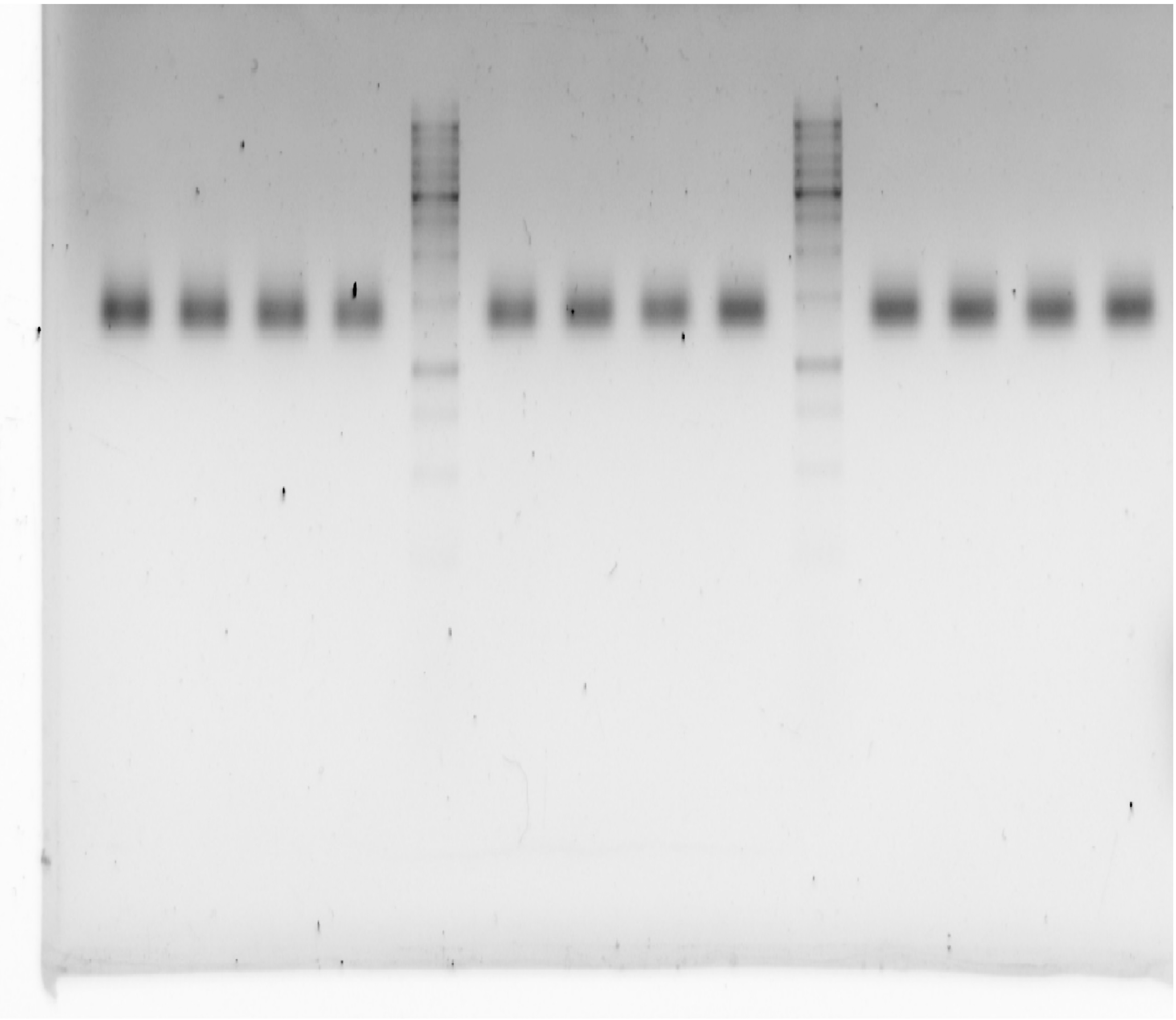

Caspase kidney

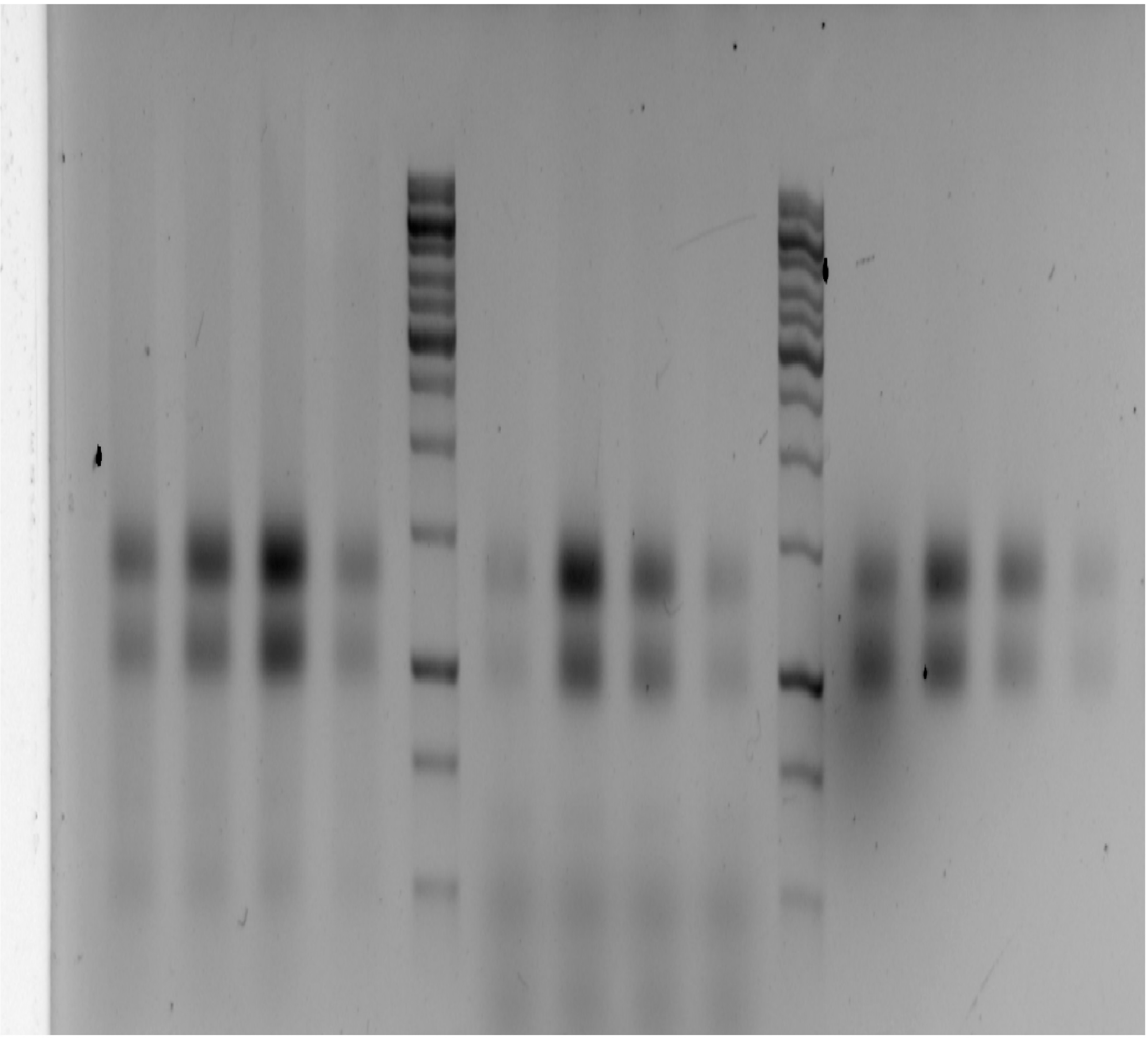

Caspase liver

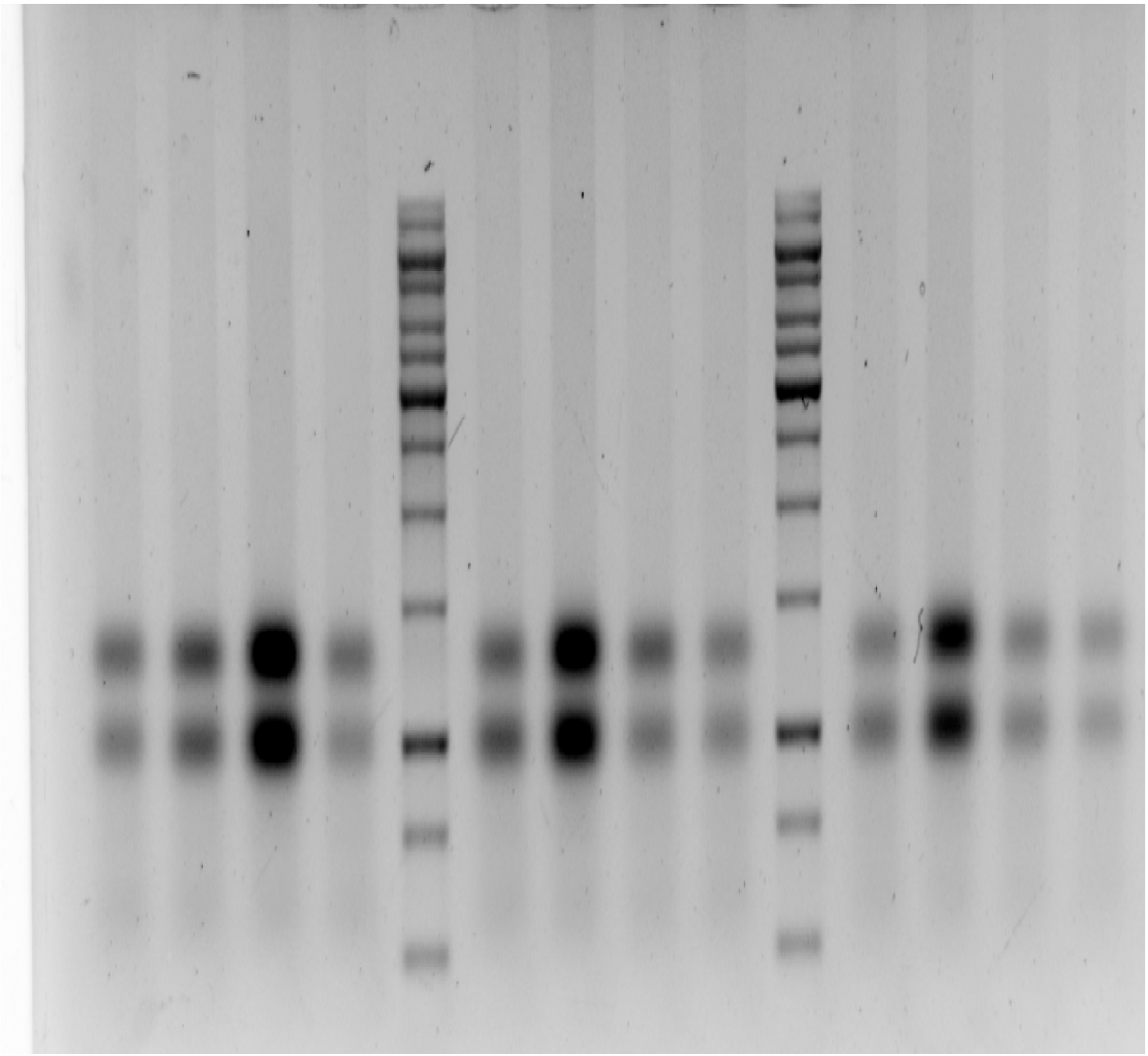

SIRT1 Kidney

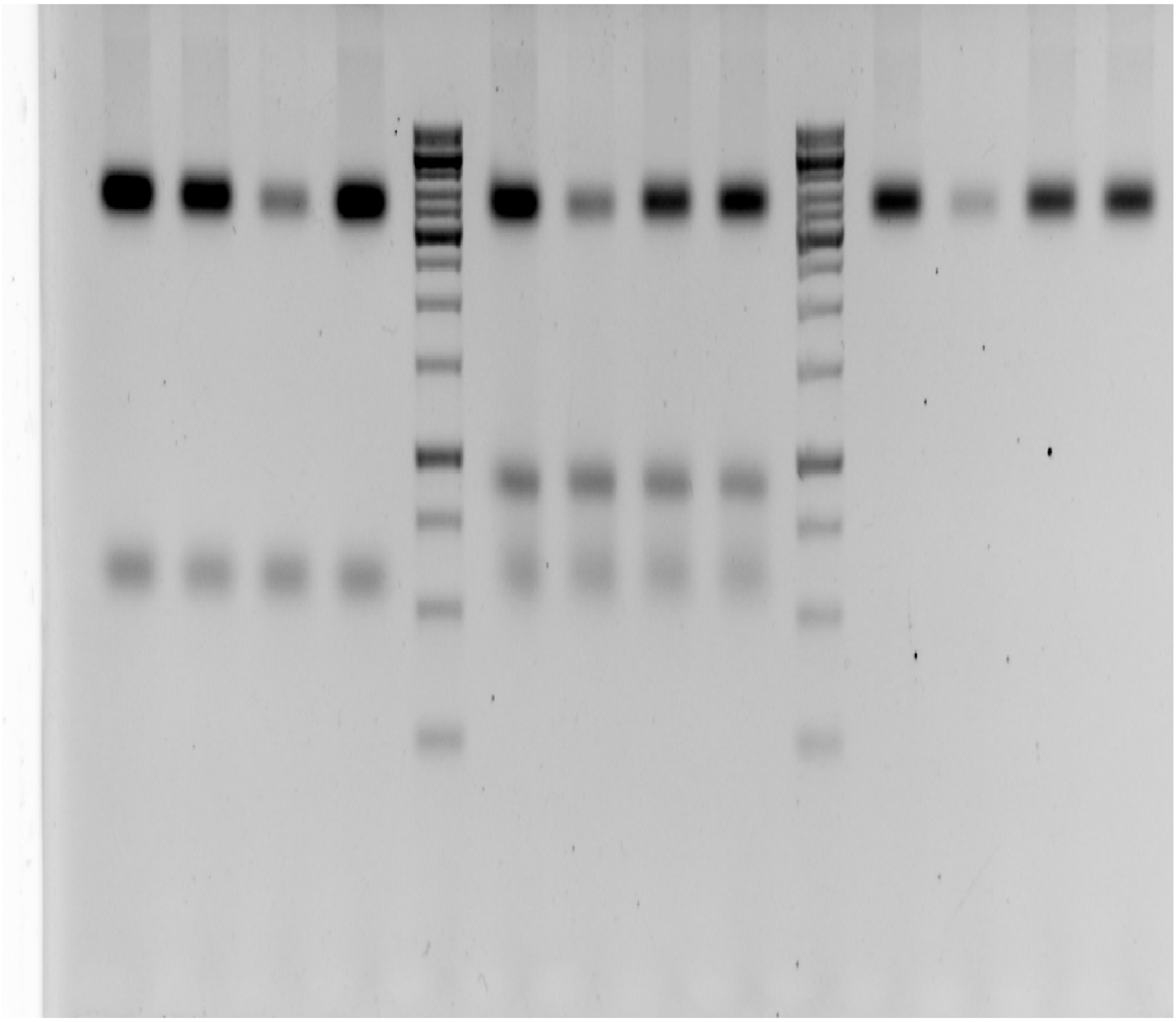

SIRT1 Liver

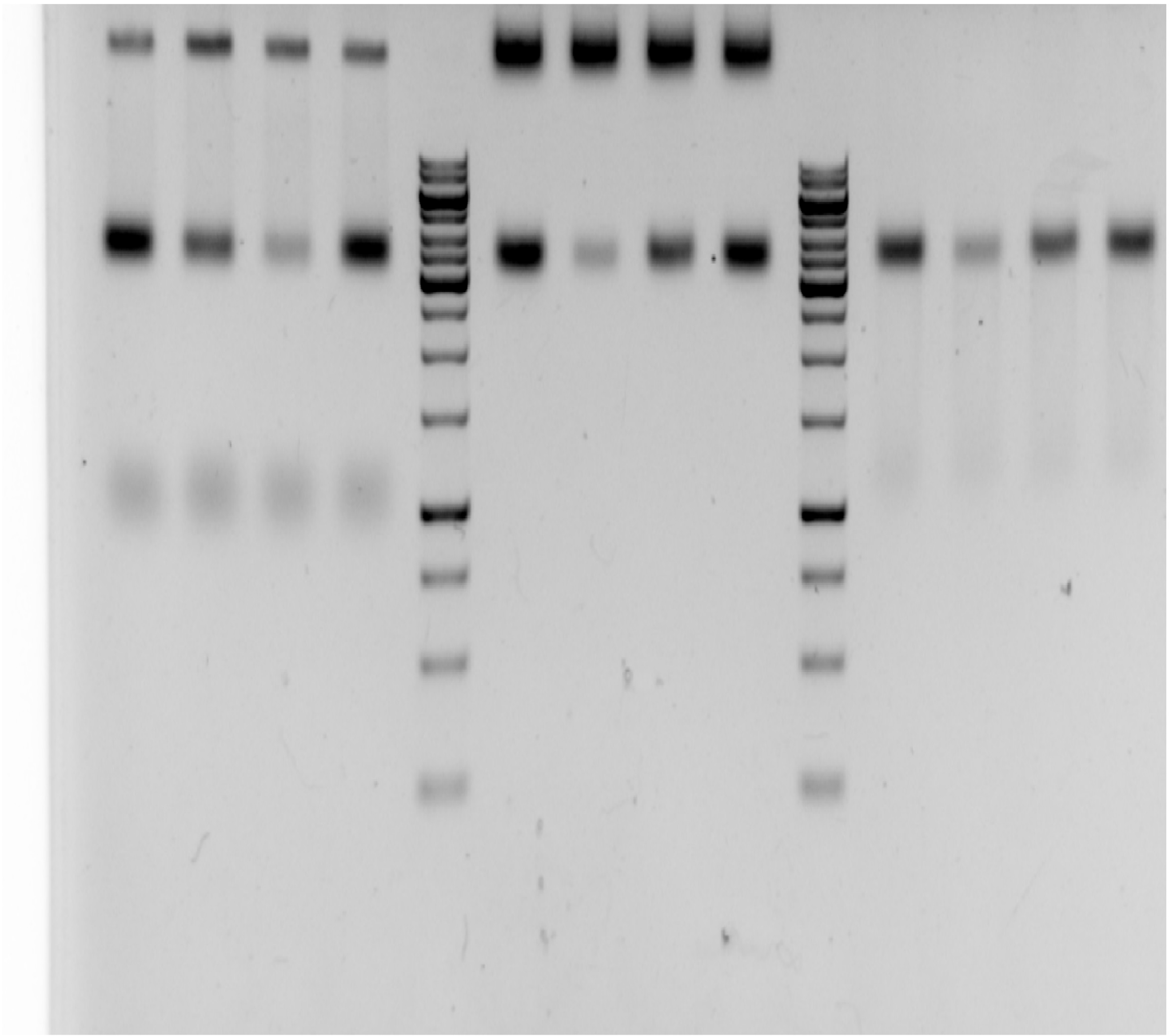

TLR4 Kidney

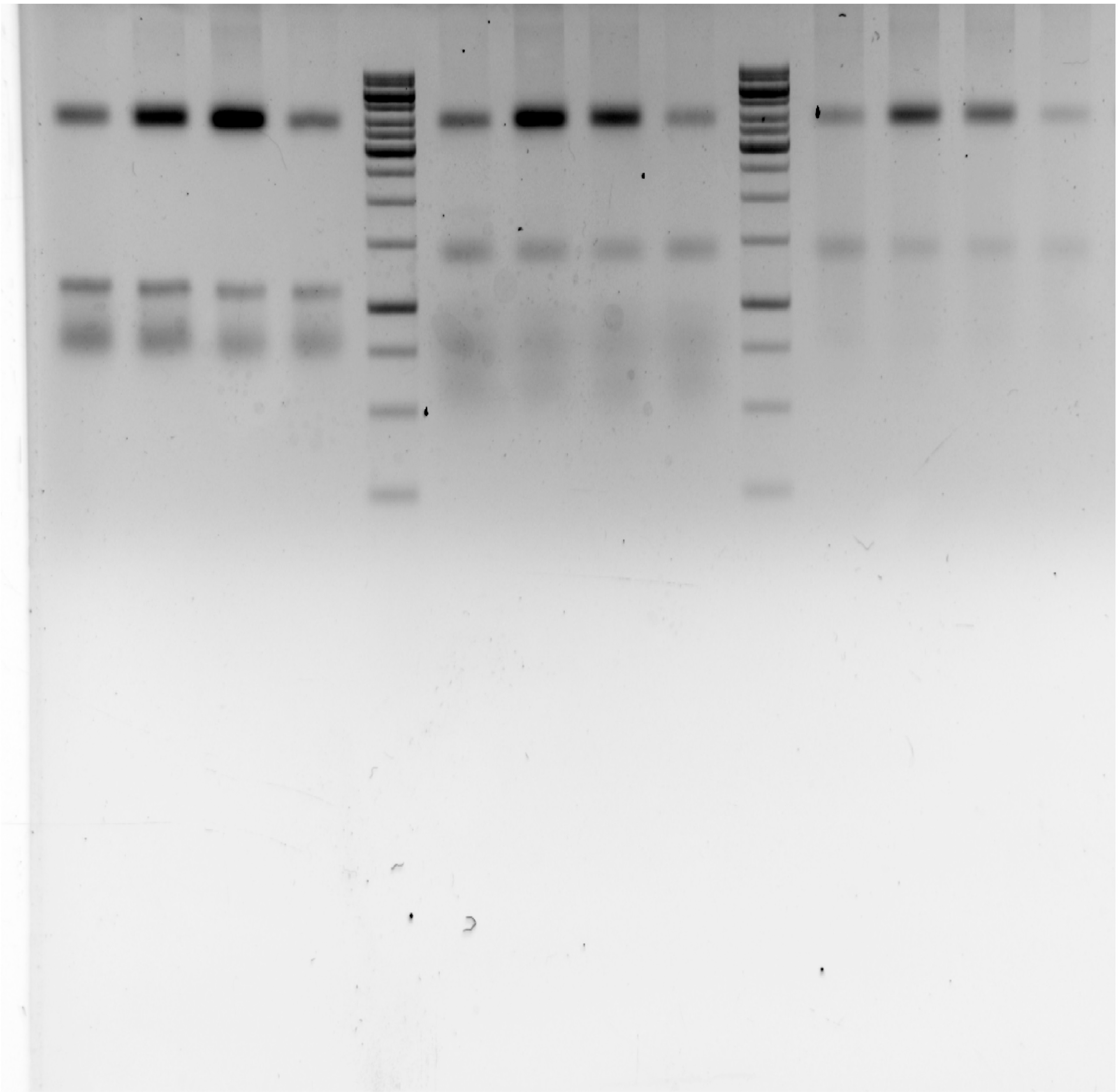

TLR4 Liver

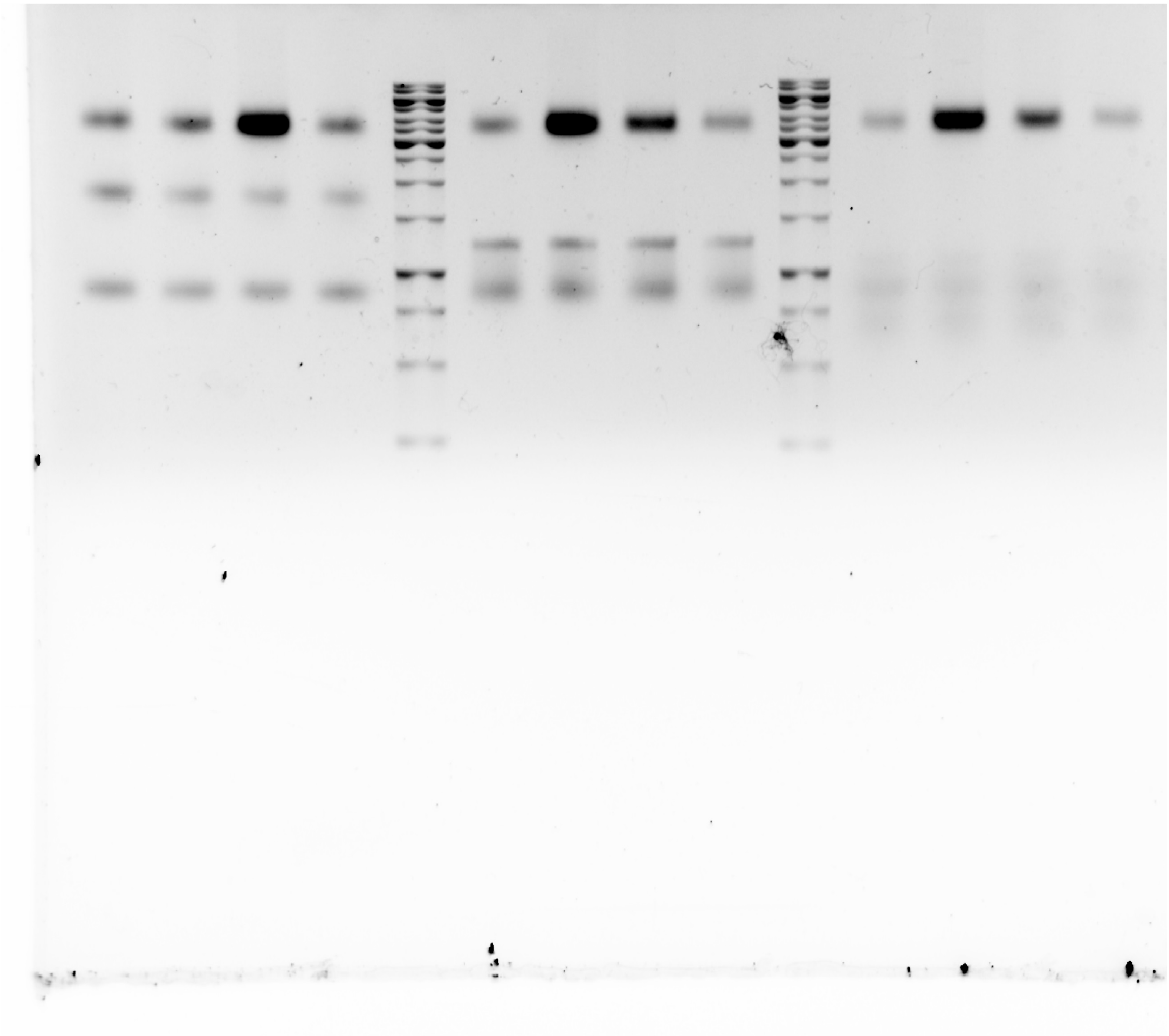

Figure 8A

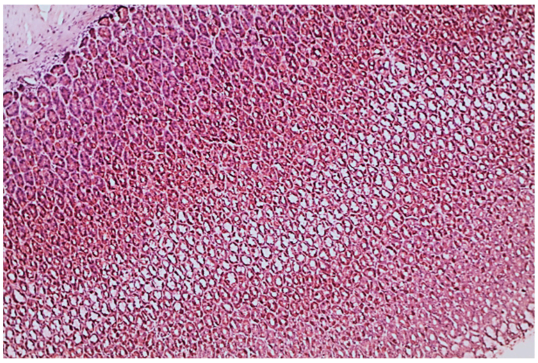

Normal control X100

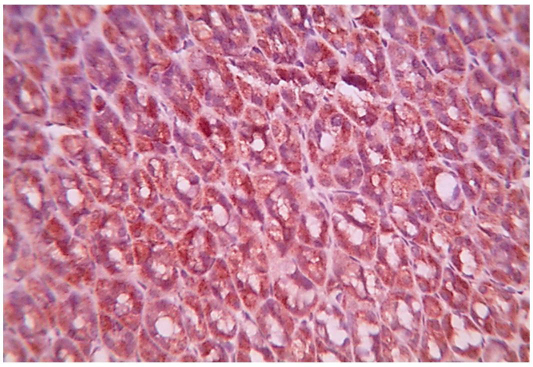

Normal control X400

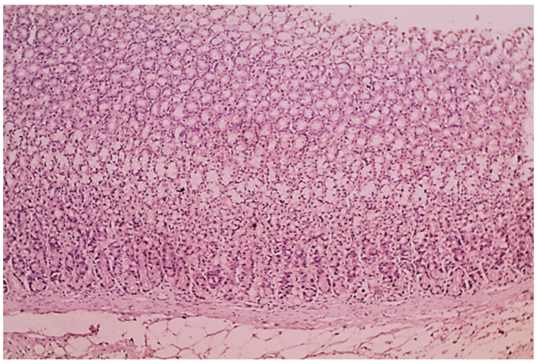

Diclofenac group X100

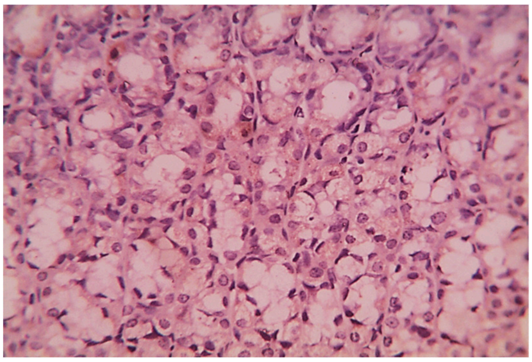

Diclofenac group X400

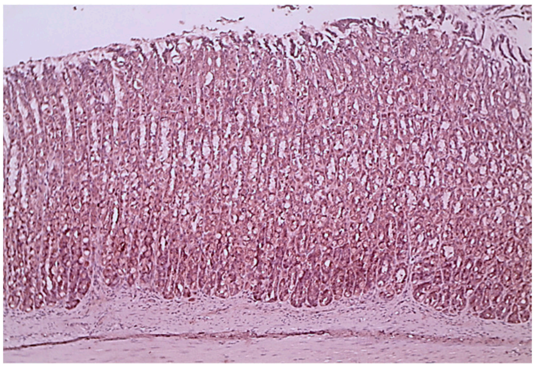

Fasudil 10 mg/kg group X100

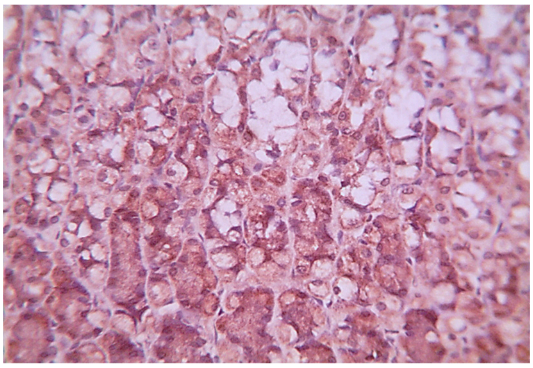

Fasudil 10 mg/kg group X400

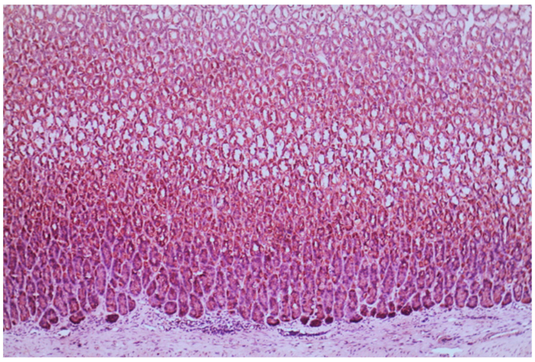

Fasudil 30 mg/kg group X100

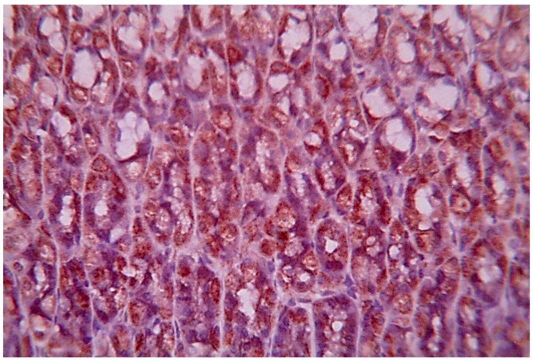

Fasudil 30 mg/kg group X400

Figure 9

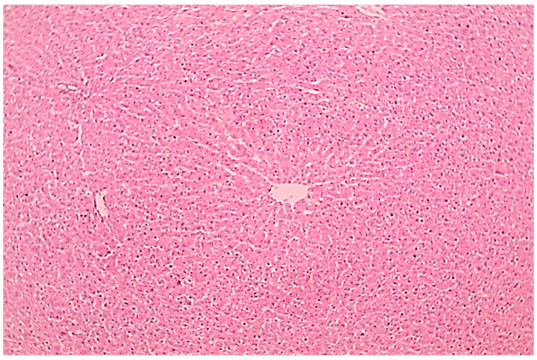

Normal Control X100

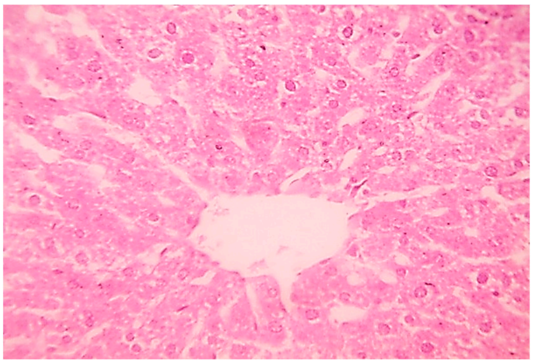

Normal control 400

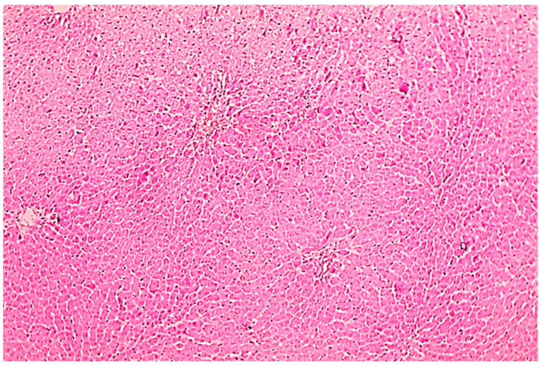

Diclofenac group X100

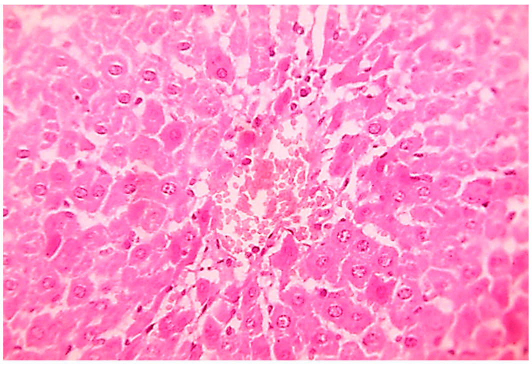

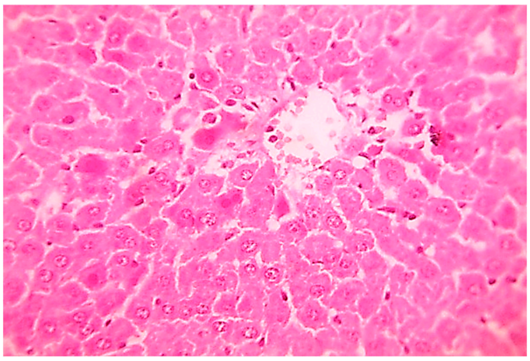

Dclofenac group X400

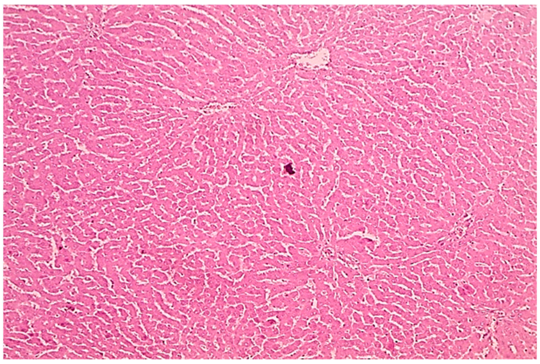

Fasudil control X100

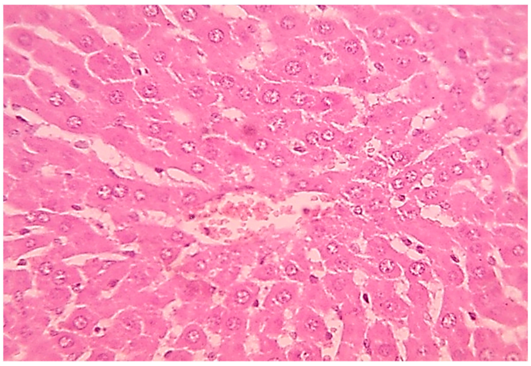

Fasudil control 400

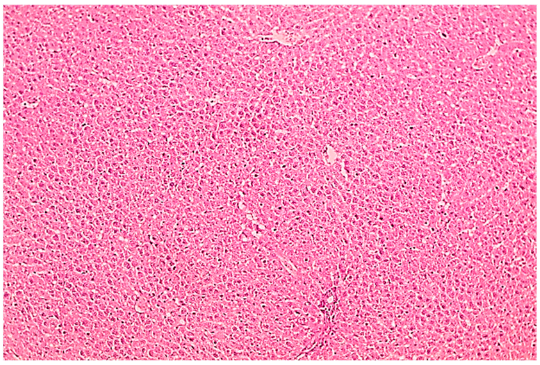

Fasudil 10 mg/kg group X100

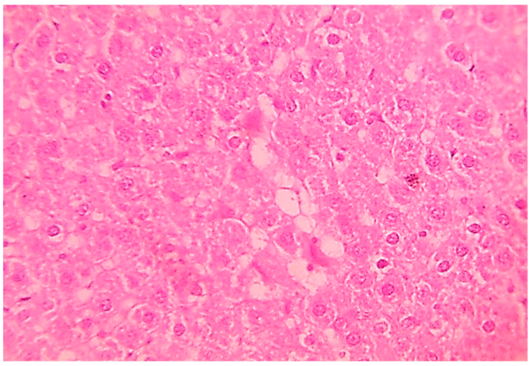

Fasudil 10 mg/kg group X400

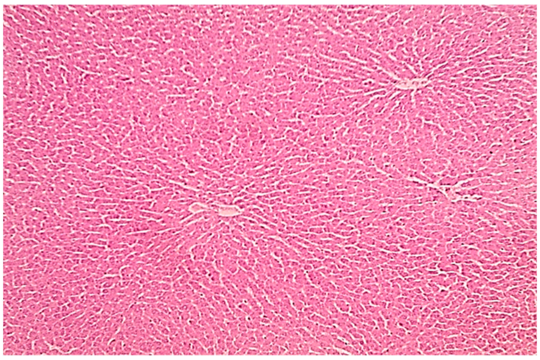

Fasudil 30 mg/kg group X100

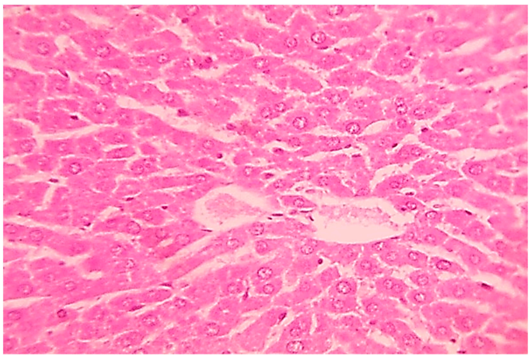

Fasudil 30 mg/kg group X400

Figure 10

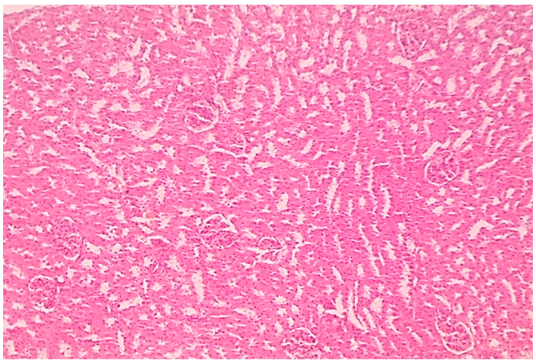

Normal control X100

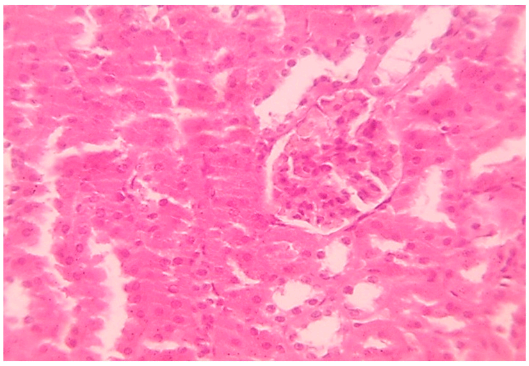

Normal control X400

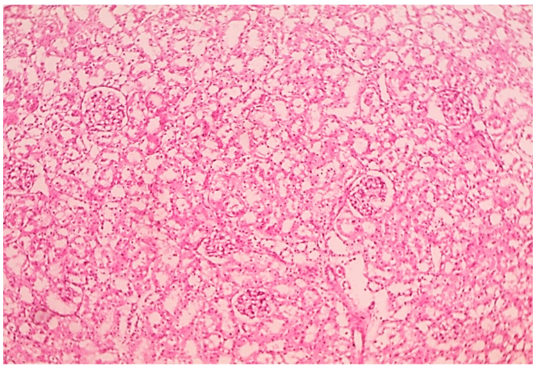

Diclofenac group X100

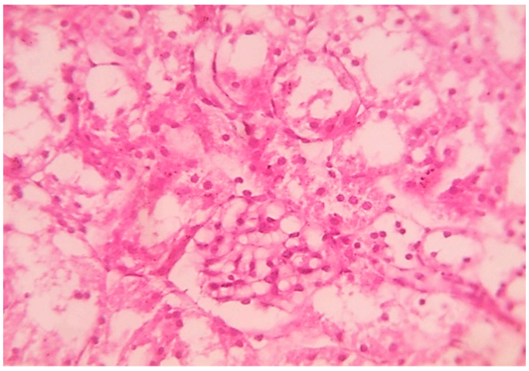

Diclofenac group X400

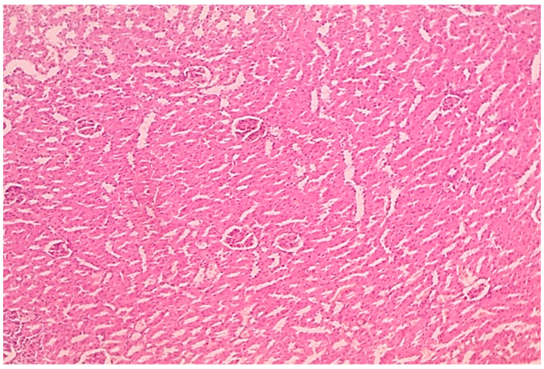

Fasudil control group X100

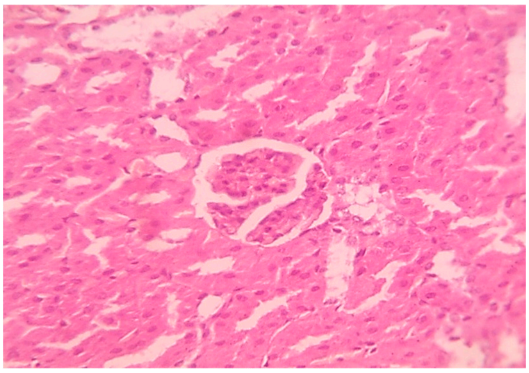

Fasudil control group X400

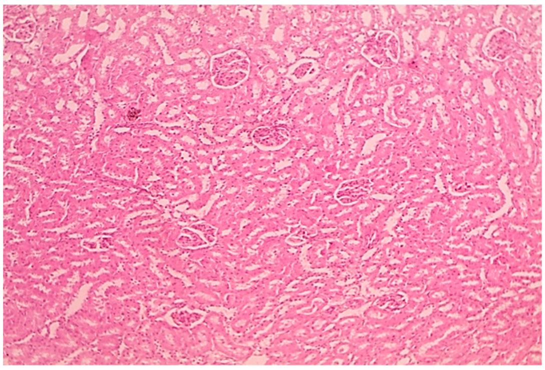

Fasudil 10 mg/kg group X100

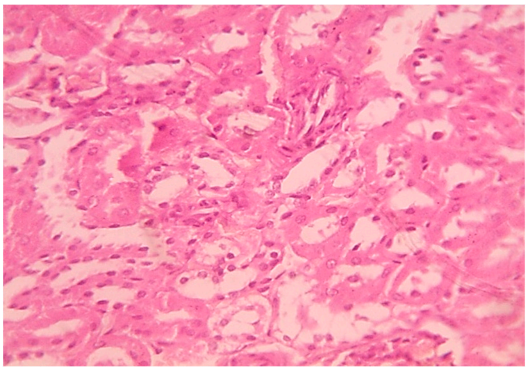

Fasudil 10 mg/kg group X400

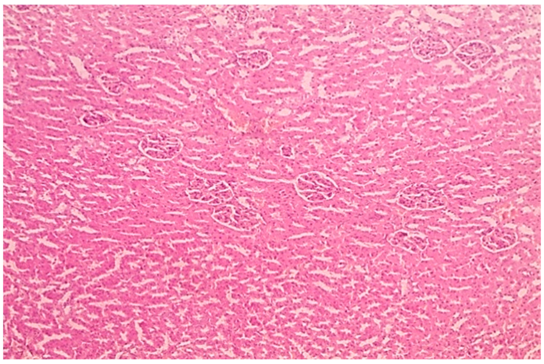

Fasudil 30 mg/kg group X100

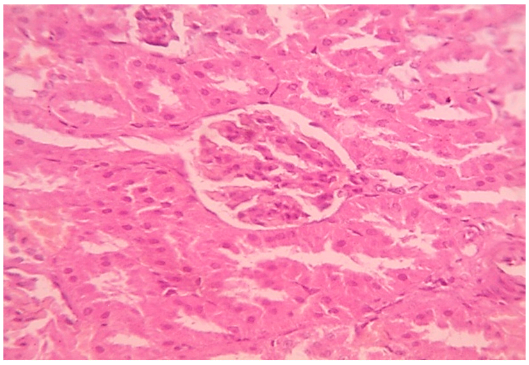

Fasudil 30 mgkg group X400

Figure 11:

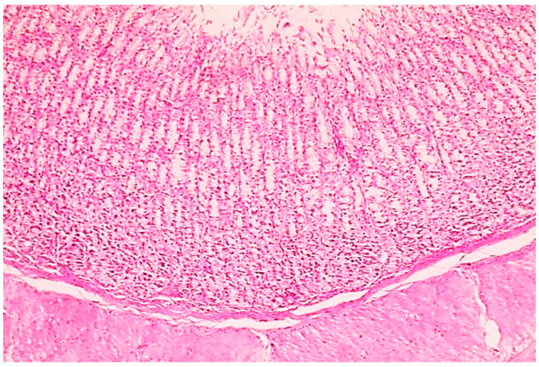

Normal control X100

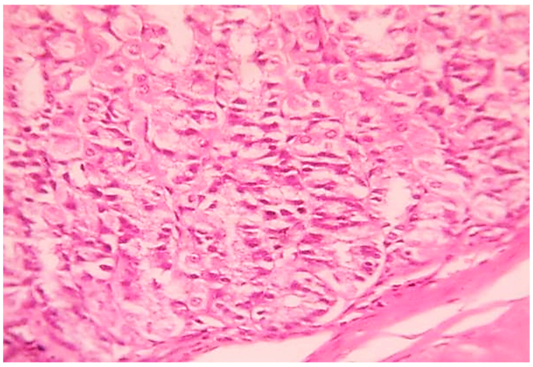

Normal control 400

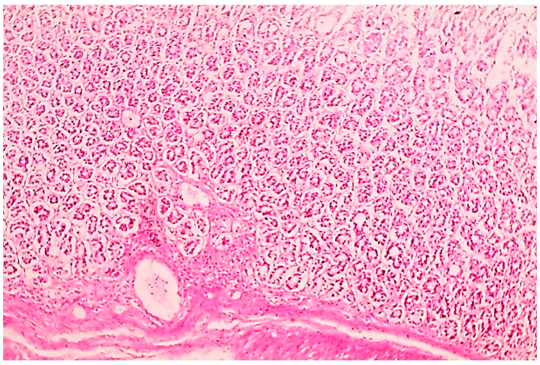
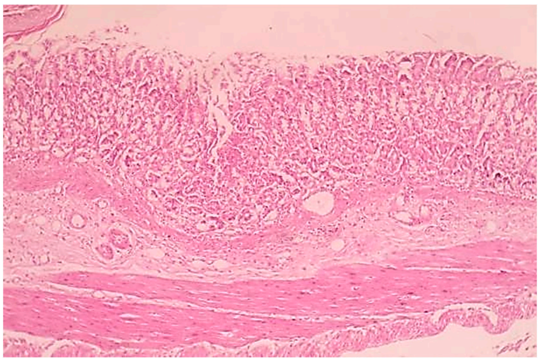

Diclofenac group X100

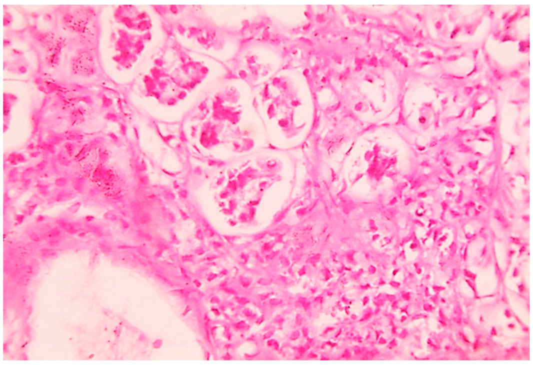
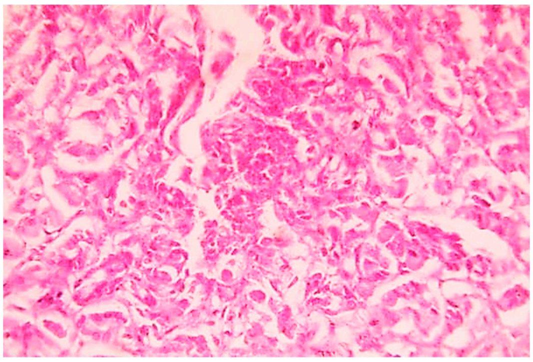

Diclofenac group X400

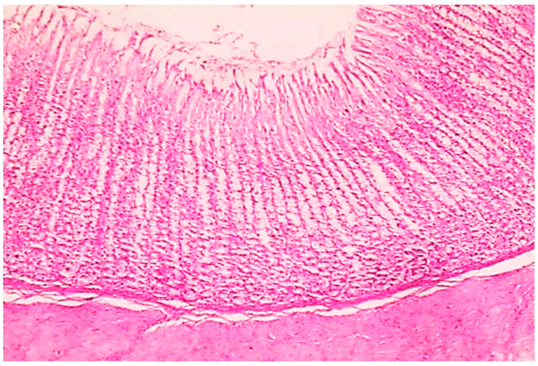

Fasudil control group X100

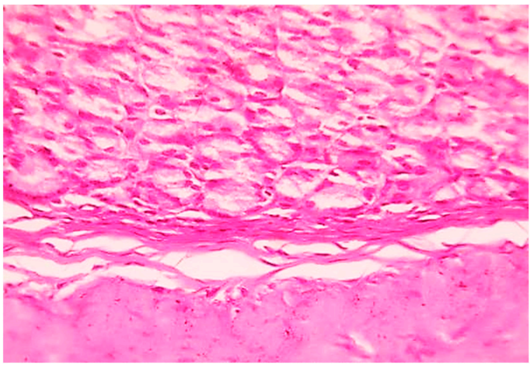

Fasudil control X400

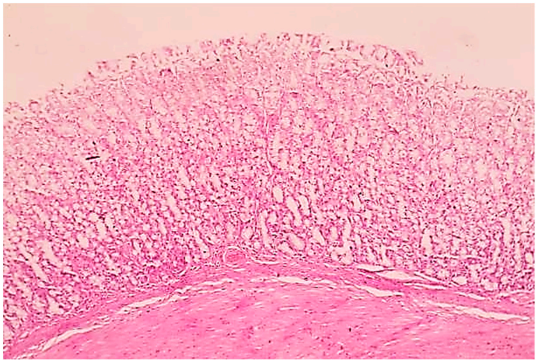

Fasudil 10 mg/kg group X100

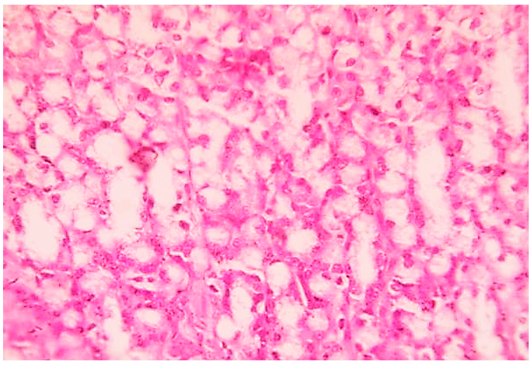

Fasudil 10 mg/kg group X400

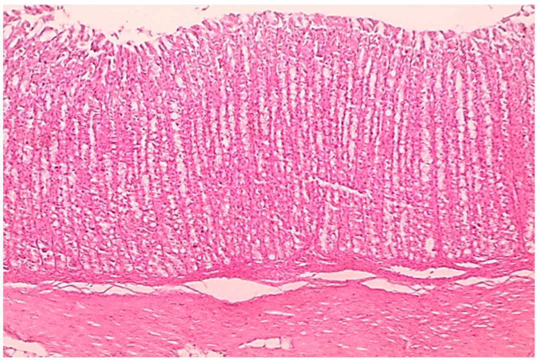

Fasudil 30 mg/kg group X100

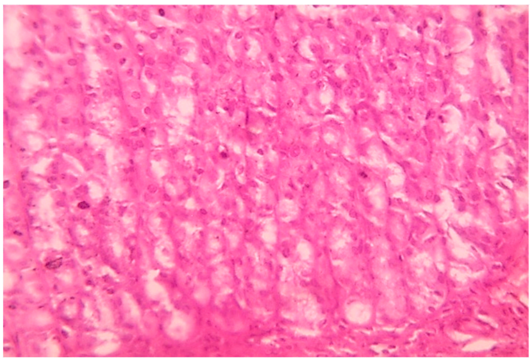

Fasudil 30 mg/kg group X400
